# Pepper mild mottle virus coat protein interacts with pepper chloroplast outer envelope membrane protein OMP24 to inhibit antiviral immunity in plants

**DOI:** 10.1093/hr/uhad046

**Published:** 2023-03-15

**Authors:** Kelei Han, Hongying Zheng, Dankan Yan, Huijie Zhou, Zhaoxing Jia, Yushan Zhai, Jian Wu, Yuwen Lu, Guanwei Wu, Shaofei Rao, Jianping Chen, Jiejun Peng, Rende Qi, Fei Yan

**Affiliations:** State Key Laboratory for Managing Biotic and Chemical Threats to the Quality and Safety of Agro-products, Institute of Plant Virology, Ningbo University, Ningbo, 315211, China; Institute of Plant Protection and Agro-Products Safety, Anhui Academy of Agricultural Sciences, Hefei, Anhui 230031, China; State Key Laboratory for Managing Biotic and Chemical Threats to the Quality and Safety of Agro-products, Institute of Plant Virology, Ningbo University, Ningbo, 315211, China; Institute of Plant Protection and Agro-Products Safety, Anhui Academy of Agricultural Sciences, Hefei, Anhui 230031, China; State Key Laboratory for Managing Biotic and Chemical Threats to the Quality and Safety of Agro-products, Institute of Plant Virology, Ningbo University, Ningbo, 315211, China; State Key Laboratory for Managing Biotic and Chemical Threats to the Quality and Safety of Agro-products, Institute of Plant Virology, Ningbo University, Ningbo, 315211, China; State Key Laboratory for Managing Biotic and Chemical Threats to the Quality and Safety of Agro-products, Institute of Plant Virology, Ningbo University, Ningbo, 315211, China; State Key Laboratory for Managing Biotic and Chemical Threats to the Quality and Safety of Agro-products, Institute of Plant Virology, Ningbo University, Ningbo, 315211, China; State Key Laboratory for Managing Biotic and Chemical Threats to the Quality and Safety of Agro-products, Institute of Plant Virology, Ningbo University, Ningbo, 315211, China; State Key Laboratory for Managing Biotic and Chemical Threats to the Quality and Safety of Agro-products, Institute of Plant Virology, Ningbo University, Ningbo, 315211, China; State Key Laboratory for Managing Biotic and Chemical Threats to the Quality and Safety of Agro-products, Institute of Plant Virology, Ningbo University, Ningbo, 315211, China; State Key Laboratory for Managing Biotic and Chemical Threats to the Quality and Safety of Agro-products, Institute of Plant Virology, Ningbo University, Ningbo, 315211, China; State Key Laboratory for Managing Biotic and Chemical Threats to the Quality and Safety of Agro-products, Institute of Plant Virology, Ningbo University, Ningbo, 315211, China; Institute of Plant Protection and Agro-Products Safety, Anhui Academy of Agricultural Sciences, Hefei, Anhui 230031, China; State Key Laboratory for Managing Biotic and Chemical Threats to the Quality and Safety of Agro-products, Institute of Plant Virology, Ningbo University, Ningbo, 315211, China

## Abstract

Pepper mild mottle virus (PMMoV) is a devastating viral pathogen of pepper (*Capsicum annuum*) but it is unclear whether and how peppers protect against PMMoV infection. The expression of the chloroplast outer membrane protein 24 (OMP24) of *C. annuum* was upregulated under PMMoV infection and it interacted with PMMoV coat protein (CP). Silencing of *OMP24* in either *C. annuum* or *Nicotiana benthamiana* facilitated PMMoV infection, whereas overexpression of *N. benthamiana OMP24* in transgenic plants inhibited PMMoV infection. Both *C. annuum* OMP24 (CaOMP24) and *N. benthamiana* OMP24 (NbOMP24) localized to the chloroplast and have a moderately hydrophobic transmembrane domain that is necessary for their localization. Overexpression of *CaOMP24* induced stromules, perinuclear chloroplast clustering, and accumulation of reactive oxygen species (ROS), the typical defense responses of chloroplasts transferring the retrograde signaling to the nucleus to regulate resistance genes. The expression of *PR1* and *PR2* was also upregulated significantly in plants overexpressing *OMP24*. Self-interaction of OMP24 was demonstrated and was required for OMP24-mediated plant defense. Interaction with PMMoV CP interfered with the self-interaction of OMP24 and impaired OMP24-induced stromules, perinuclear chloroplast clustering and ROS accumulation. The results demonstrate the defense function of OMP24 in pepper during viral infection and suggest a possible mechanism by which PMMoV CP modulates the plant defense to facilitate viral infection.

## Introduction

Pepper mild mottle virus (PMMoV), a member of the genus *Tobamovirus*, is the major viral pathogen of pepper (*Capsicum annuum* L.), resulting in significant crop losses around the world [1, 48]. PMMoV is transmitted directly to plants by infected sap and also through contaminated seed. PMMoV has a single-stranded RNA genome (about 6.4 Kb) that encodes four proteins: p126 (viral suppressor of RNA silencing), p183 (RNA-dependent RNA polymerase), movement protein, and coat protein (CP) [4]. The CP of all tobamoviruses is encoded by a subgenomic RNA and plays a versatile role during the viral infection process. Besides encapsidating the viral genome, it is involved in systemic virus movement, cross-protection, pathogenicity, and symptom development [55]. Changes in the PMMoV CP amino acid sequence are reported to be responsible for breaking the *L^3^* and *L^4^* mediated-resistance of the host [6, 24]. Our previous study showed that the 20th amino acid of PMMoV CP determined the chlorosis symptom and is responsible for subcellular localization to the chloroplast [25]. However, the detailed biological function of the relationship between PMMoV CP and chloroplasts has not been determined.

In addition to supplying the plant with energy, chloroplasts are the source of defense signals including phytohormones, reactive oxygen species (ROS) and calcium (Ca^2+^), hence playing critical roles in plant immunity [36, 42]. Moreover, as an environmental sensor, chloroplasts communicate with the nucleus to change the expression of thousands of proteins, which is termed retrograde signaling [15]. Perinuclear chloroplast clustering (PCC) and the formation of stromules that are dynamic tubular extensions from chloroplasts are general plant immune responses of plants under stresses [13, 16, 17, 42]. The movement of chloroplasts to the nucleus is believed to be guided by stromules. A retrograde signal such as ROS originating in the chloroplasts is transferred to the nucleus through the stromules connecting the chloroplast to the nucleus, regulating resistance gene expression and thereby mediating plant immunity [26, 27].

Chloroplasts have a double-membrane structure. The outer envelope membrane is a biochemical and physical barrier and the outer membrane proteins (OMPs) play important roles in intracellular communication and organelle biogenesis [5, 21, 28, 29, 31]. In addition, chloroplast outer membrane proteins DGD1, EDS5, and JASSY have been shown to participate in the biosynthesis of the lipid digalactosyldiacylglycerol (DGDG), SA and jasmonate, respectively [23, 41, 56]. It is not known if and how the OMPs participate in plant defense.

**Figure 1:**
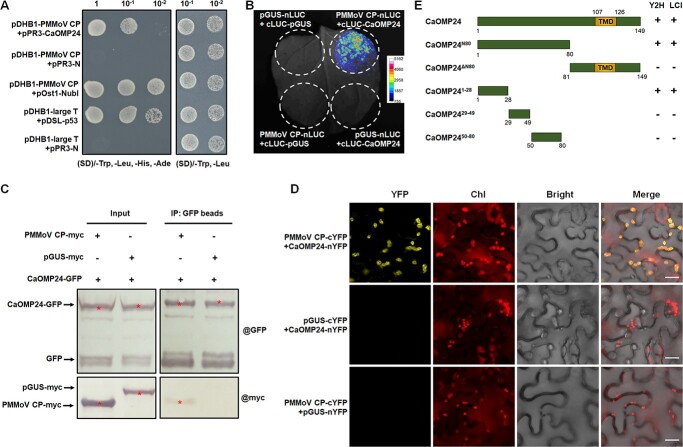
PMMoV CP interacts with CaOMP24. **A** PMMoV CP interacted with CaOMP24 in a yeast two hybrid (Y2H) assay. Yeast NMY51 co-transformed with pDHB1-large T + pDSL-p53, pDHB1-PMMoV CP + pOst1-NubI was the positive control; pDHB1-large T + pPR3-N, pDHB1-PMMoV CP + pPR3-N were the negative controls. Serial 10-fold dilutions of yeast cells were plated on selective medium (SD)/−Trp, −Leu, -His, −Ade or (SD)/−Trp, −Leu for 3–5 days. **B** Split-luciferase complementation (LCI) assays demonstrating that PMMoV CP interacts with CaOMP24 in *N. benthamiana* leaves. The N- or C-terminal fragments of luciferase (LUC) were fused to the C-terminus of PMMoV CP and N-terminus of CaOMP24. β-glucuronidase (GUS) was used as a negative control. **C** Co-IP analysis of the interaction between PMMoV CP and CaOMP24. *N. benthamiana* leaves transiently co-expressing CaOMP24-GFP with PMMoV CP-Myc or pGUS-Myc were harvested at 60 hpi. Total proteins were immunoprecipitated with anti-GFP beads and samples before (Input) and after (IP) immunopurification were detected by western blot with an anti-GFP or anti-Myc antibody. The red asterisks indicate the expected band sizes. **D** BiFC analysis of the interaction between PMMoV CP and CaOMP24. The N- (nYFP) or C-terminal (cYFP) fragments of YFP were fused to the C-terminus of PMMoV CP and CaOMP24. pGUS was used as a negative control. Confocal analysis was performed at 2 dpi. Scale bars, 20 μm. **E** Summary of interactions between PMMoV CP and the CaOMP24 truncated mutants as determined by Y2H and LIC.

Here, we identified that the chloroplast OMP24 has a defense role in pepper during PMMoV infection. Overexpression of OMP24 caused stromules, PCC, and accumulation of ROS, as well as the upregulated expression of resistance genes. PMMoV CP interacts with OMP24 and interferes with the self-interaction of OMP24 and hence inhibits the OMP24-mediated resistance to facilitate PMMoV infection. These results provide the first evidence for a mechanism by which OMPs are involved in plant defense against viral infection.

## Results

### PMMoV CP interacts with CaOMP24

To identify possible host proteins required for PMMoV CP to perform its function in viral infection, a cDNA library of *C. annuum* was used for protein screening with PMMoV CP as the bait by a split-ubiquitin membrane yeast two-hybrid DUALhunter system. One of the candidate proteins gave a positive signal on the selective media (SD/−Trp-Leu-His-Trp) (Fig. 1A). The prey fragment in this possible interaction was sequenced with primers CYC1-F/R. Result showed that it covered 84% of the Capana12g002319 CDS in the *C. annuum* database (https://solgenomics.net/ftp/genomes/Capsicum_annuum/). Capana12g002319 contains an ORF of 450 nucleotides predicted to encode a 149-amino acid protein that has 30.2% identity to chloroplast OMP24 in spinach (*Spinacia oleracea* L.) (Fig. S1, see online supplementary material). We therefore named Capana12g002319 as CaOMP24. OMP24 in spinach is reported to be an acidic protein (calculated isoelectric point 4.8) deeply embedded in the chloroplast outer membrane [18]. Alignment of protein sequences from *C. annuum, Nicotiana benthamiana, Arabidopsis thaliana* and *S. oleracea* showed that OMP24 has a conserved motif near its C-terminus (Fig. S1, see online supplementary material).

**Figure 2:**
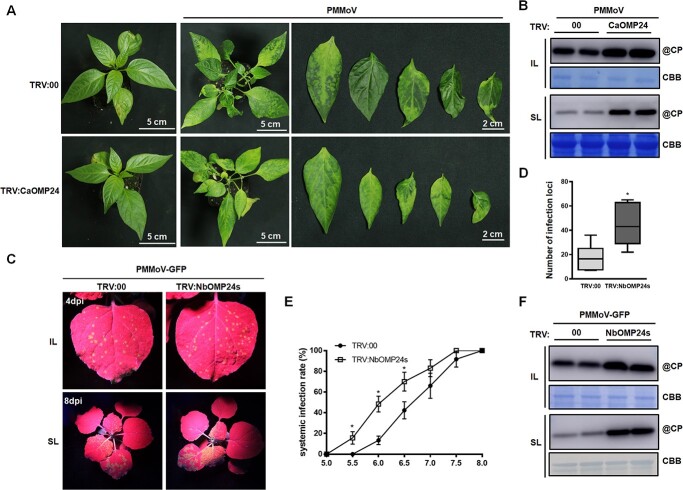
Silencing of *OMP24* facilitates PMMoV infection in pepper and *N. benthamiana*. **A** Silencing of *CaOMP24* promoted the infection of PMMoV in pepper. TRV-induced gene silencing of *CaOMP24* showed no obvious phenotype in pepper at 18 dpi (left). Typical viral symptoms on the systemic leaves 30 d after inoculation with PMMoV are shown (right). **B** The accumulation of PMMoV CP in inoculated leaves (IL, 5 dpi) and systemically infected leaves (SL, 30 dpi) of TRV:00 and TRV:CaOMP24 plants as detected by western blot using PMMoV CP polyclonal antiserum. Rubisco large subunit (RbcL) was used as loading control. **C** GFP fluorescence of PMMoV-GFP infection in inoculated and systemic leaves at 4 dpi and 8 dpi under UV light. **D** Number of infection foci on inoculated leaves (IL) 4 days after PMMoV-GFP infection. Error bars show the mean ± SD of three replicates (at least 16 plants per replicate). **E** Systemic infection rates at different times after PMMoV-GFP inoculation. Error bars show the mean ± SD of three replicates (at least 16 plants per replicate). **F** Western blot showing the increased accumulation of PMMoV CP in IL (upper) and SL (lower) of TRV:NbOMP24s plants compared to TRV:00.

Further Y2H assays confirmed the interaction of CaOMP24 with PMMoV CP (Fig. 1A). Split-luciferase complementation imaging (LCI), bimolecular fluorescence complementation (BiFC) and Co-IP experiments were further performed to verify the interaction. For the LCI assay, we constructed PMMoV CP-nLUC (with nLUC tagged at the C-terminus of PMMoV CP), cLUC-CaOMP24 (with cLUC tagged at the N-terminus of CaOMP24). A partial sequence of β-glucuronidase (pGUS) with 200 amino acids was used as a negative control. The co-expression of cLUC-CaOMP24 and PMMoV CP-nLUC in *N. benthamiana* leaves via Agrobacterium infiltration produced a bioluminescence signal at 2 days post inoculation (dpi), while no bioluminescence was detectable in the combinations cLUC-CaOMP24 + pGUS-nLUC, PMMoV CP-nLUC + cLUC-pGUS and pGUS-nLUC + cLUC-pGUS, confirming the interaction between CaOMP24 and PMMoV CP (Fig. 1B). In a CoIP assay, plasmids encoding CaOMP24-GFP (C-terminal tagging with GFP) and PMMoV CP-Myc (C-terminal tagging with 4 × Myc) were co-transiently expressed in *N. benthamiana* leaves, with pGUS-Myc used as a negative control. Total protein was extracted at 60 hours post inoculation (hpi), immunoprecipitated using anti-GFP beads, and coprecipitated proteins were probed with an anti-Myc antibody. CaOMP24-GFP co-immunoprecipitated with PMMoV CP-Myc, but not with pGUS-Myc (Fig. 1C). In the BiFC assay, YFP fluorescence was observed in the combination PMMoV CP-cYFP and CaOMP24-nYFP (Fig. 1D). These results demonstrated that PMMoV CP interacted with CaOMP24 in planta.

To analyse the key region of CaOMP24 that interacted with PMMoV CP, we divided CaOMP24 into two peptides (N-terminal N80 and C-terminal ΔN80). Both Y2H and LCI experiments confirmed that CaOMP24^N80^ but not CaOMP24^ΔN80^ could interact with PMMoV CP (Fig. S2B and C, see online supplementary material). To better define the region of CaOMP24 required for the interaction, we used a series of truncated mutants of CaOMP24 with 1st–28th, 29th–49th or 50th–80th amino acids for analysis. The mutant CaOMP24^1–28^ interacted with PMMoV CP, but there was no interaction with either CaOMP24^29–49^ or CaOMP24^50–80^ (Fig. S2B and C, see online supplementary material ), indicating that the N-terminal 28 amino acid region of CaOMP24 is necessary for its interaction with PMMoV CP.

### Silencing of *OMP24* facilitates PMMoV infection in pepper and *N. benthamiana*

To investigate the function of CaOMP24 in pepper plants during PMMoV infection, the tobacco rattle virus (TRV)-induced gene silencing (VIGS) system was used to silence *CaOMP24* and the plants were then inoculated with PMMoV. For analysis, a partial sequence of CaOMP24 was inserted into RNA2 of TRV, producing TRV:CaOMP24. At 18 dpi of TRV:CaOMP24 inoculation, the expression of *CaOMP24* was decreased to ~30% of the normal level in the control inoculated with the empty TRV (TRV:00), while the plants did not show any obvious phenotype (Fig. 2A; Fig. S3A, see online supplementary material). Plants were then rub-inoculated with PMMoV. At 30 dpi of PMMoV infection, the silenced plants had more severe disease symptoms than the control with more curling and shrinking leaves (Fig. 2A). Consistently, PMMoV CP accumulated at a higher level in the silenced plants (Fig. 2B).

We also did similar experiments in *N. benthamiana,* a model plant for studying virus-host interactions [22]. There are two *CaOMP24* homologues in *N. benthamiana* with 96.7% nucleotide identity and 95.0% amino acid identity, namely *NbOMP24.1* (Niben101Scf02622g10012) and *NbOMP24.2* (Niben101Scf05697g03010). Both NbOMP24.1 and NbOMP24.2 interacted with PMMoV CP in Y2H, LIC, and BIFC experiments (Fig. S4, see online supplementary material). We investigated the role of *NbOMP24s* in PMMoV infection using the TRV-VIGS system. Due to their high sequence identity, *NbOMP24.1* and *NbOMP24.2* would be silenced simultaneously in VIGS analysis. Silencing of *NbOMP24s* decreased the transcript level of *NbOMP24s* to 20% at 12 dpi but did not cause obvious developmental change in the plants (Fig. S3B and C, see online supplementary material). Plants were then rub-inoculated with PMMoV-GFP. Compared to TRV:00-treated plants, more fluorescent foci appeared on the inoculated leaves of *NbOMP24*-silenced plants at 4 dpi of PMMoV infection (Fig. 2C and D). Moreover, the speed of systemic infection was remarkably faster in *NbOMP24s*-silenced plants than in the non-silenced ones (Fig. 2E). The accumulation of PMMoV CP in both inoculated leaves (IL) and systemically infected leaves (SL) of silenced plants was significantly higher than that in the non-silenced controls (Fig. 2F).

To eliminate any effect of TRV on PMMoV infection, transient gene silencing experiments were done to confirm the effect of silencing *NbOMP24s* on PMMoV infection. The left and right sides of a single *N. benthamiana* leaf were infiltrated with agrobacterium strain GV3101 containing the construct expressing hairpin RNA of *NbOMP24* (hpNbOMP24) and hpGUS, respectively, and then inoculated with PMMoV-GFP. Results of qRT-PCR showed that the expression level of *NbOMP24s* was reduced to 28% of the control (Fig. S5B, see online supplementary material). The *NbOMP24s*-silenced regions had more fluorescent spots than the controls under UV light at 3.5 dpi (Fig. S5A, see online supplementary material). Correspondingly, PMMoV CP accumulated at higher levels in *NbOMP24*-silenced regions than in non-silenced regions (Fig. S5C, see online supplementary material).

**Figure 3:**
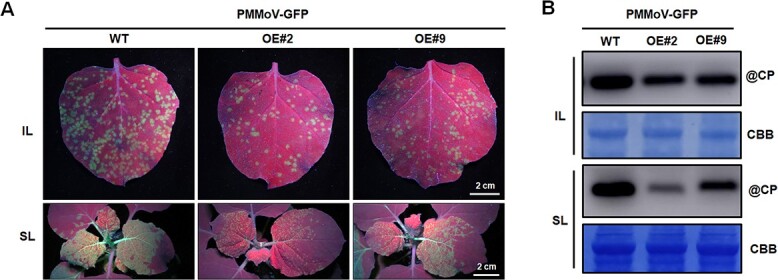
Overexpression of NbOMP24.1 in transgenic *N. benthamiana* enhances resistance to PMMoV. **A** GFP fluorescence and viral symptoms in OE-NbOMP24.1 plants inoculated with PMMoV-GFP. Inoculated and systemic leaves were photographed under UV light at 4 dpi and 7 dpi. **B** Western blotting results showing the accumulation of PMMoV CP in the inoculated leaves (IL) and systemically infected leaves (SL) from OE-NbOMP24.1 plants. Total protein was extracted from PMMoV-GFP-inoculated leaves at 4 dpi or systemically infected leaves at 7 dpi.

Taken together, these results demonstrate that silencing of *OMP24* facilitated PMMoV infection in both pepper and *N. benthamiana*.

### Overexpression of *NbOMP24.1* suppresses PMMoV infection on *N. benthamiana*

The expression of *CaOMP24* and *NbOMP24s* was up-regulated in plants during PMMoV infection (Fig. S6A and B, see online supplementary material) but expression of CP alone did not affect the expression of *NbOMP24s* (Fig. S6C, see online supplementary material). We also detected the response of *NbOMP24s* to seven other pepper-infecting viruses*.* Three of the seven viruses tested caused an up-regulation in the expression of NbOMP24s, while the remaining four viruses had no effect on the expression of NbOMP24s (Fig. S6D, see online supplementary material), which suggests that there may be some specificity in the interactions between the viruses and the plant host.

**Figure 4:**
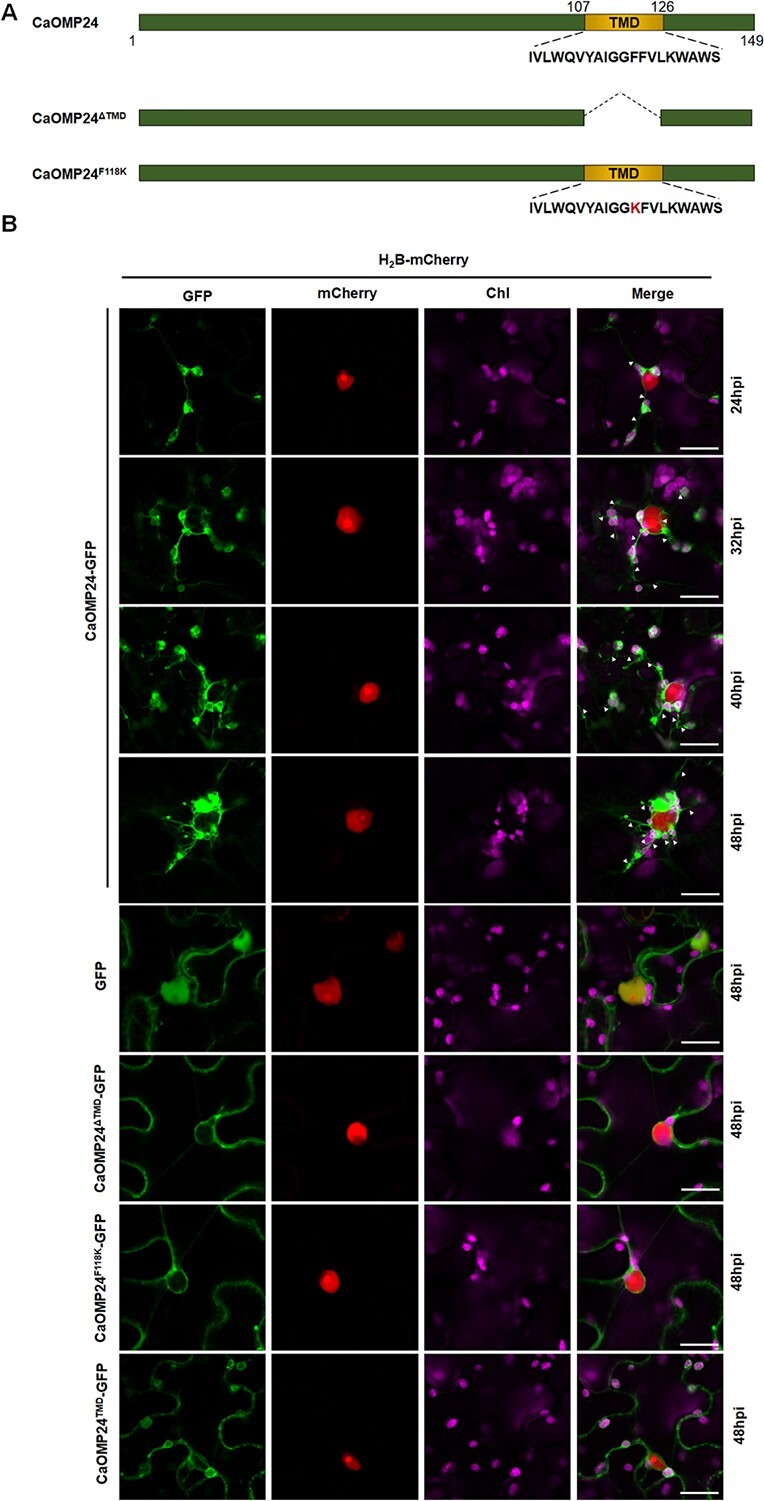
Expression of CaOMP24 induces chloroplast stromules and perinuclear chloroplast clustering. **A** Schematic representation of CaOMP24 and its mutants. TMD, transmembrane domain. **B** Co-localization study of CaOMP24 and its mutants with the nuclear maker histone H2B. Line 1–4: Time course based confocal microscopic analysis of *N. benthamiana* leaves after Agrobacterium infiltration of CaOMP24-GFP and H2B-mCherry. Line 5–8: Confocal microscopy images of *N. benthamiana* epidermal leaf cells expressing GFP, CaOMP24^ΔTMD^-GFP, CaOMP24^F118K^-GFP, CaOMP24^TMD^-GFP with H2B-mCherry at 48 hours after infiltration (hpi). H2B-mCherry served as a nuclear marker. GFP fluorescence (GFP) in green, mCherry fluorescence (mCherry) in red, chlorophyll autofluorescence (Chl) in magenta. Scale bars, 20 μm. Triangles point to the stromules.

To further confirm the roles of OMP24 in defense against PMMoV, transgenic *N. benthamiana* plants expressing Myc-tagged *NbOMP24.1* driven by the cauliflower mosaic virus (CaMV) 35S promotor were generated. The expression of NbOMP24.1-myc in transgenic plants was confirmed by western blot, and the transgenic plants had a similar phenotype to wild-type (Fig. S7, see online supplementary material). Two independent transgenic lines (designated as OE#2 and OE#9) were chosen for viral challenge. At 4 dpi of PMMoV-GFP infection, the numbers of infection foci on the inoculated leaves of OE#2 and OE#9 plants were less than on the wild-type plants (Fig. 3A). At 7 dpi, the fluorescence associated with systemic infection of transgenic plants was extensive (Fig. 3A). The accumulation of PMMoV CP was less in both the inoculated and systemic leaves of transgenic plants than in control plants (Fig. 3B). The results further demonstrate the defense role of OMP24 against PMMoV infection.

### CaOMP24 localizes to the chloroplast and induces stromules and PCC

**Figure 5:**
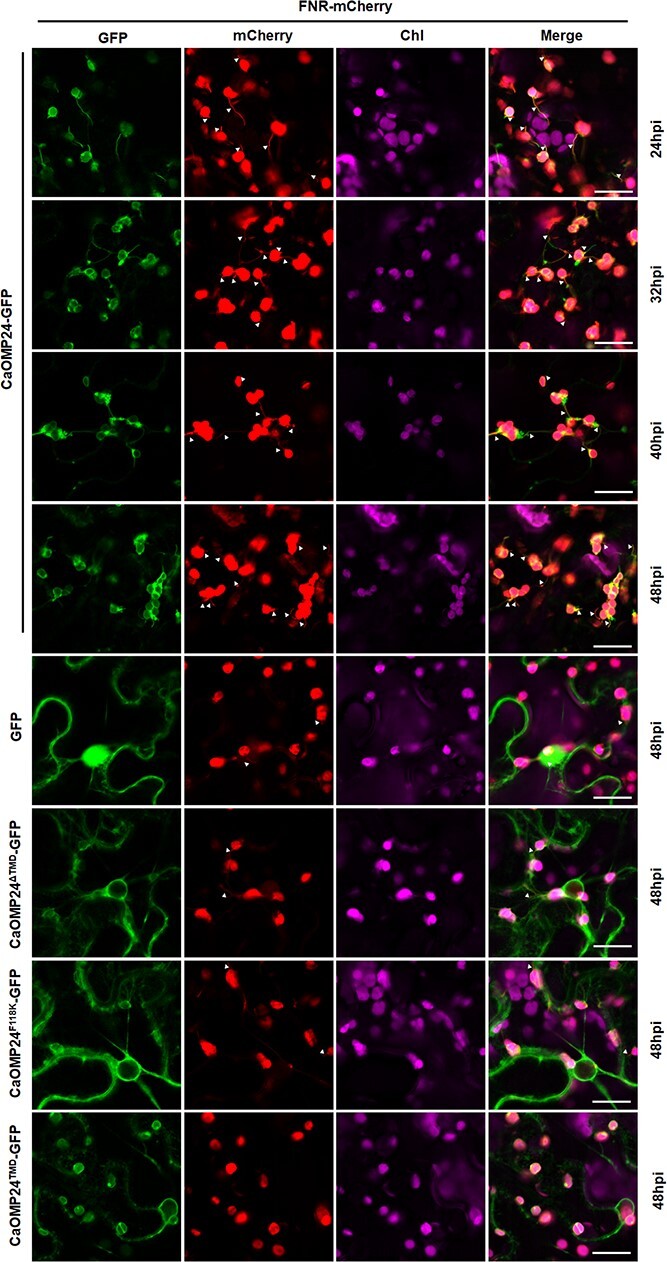
Co-localization study of CaOMP24 and its mutants with the stromule marker FNR. Confocal microscopy images of *N. benthamiana* leaf epidermal cells co-expressing FNR-mCherry (stromule marker) with CaOMP24-GFP, CaOMP24^∆^™^D^-GFP, CaOMP24^F118K^-GFP, and CaOMP24™^D^-GFP. GFP was used as a control. Scale bars, 20 μm. Triangles point to the stromules.

To understand the possible mechanism by which OMP24 defends against PMMoV infection, we first investigated its chloroplast localization and its effect on chloroplasts in epidermal cells of *N. benthamiana*. The complete open reading frame of *CaOMP24* was cloned into the pCV-N-GFP vector (CaOMP24-GFP) to express CaOMP24 with a C-terminal GFP tag. CaOMP24-GFP was then expressed in *N. benthamiana* leaves by agroinfiltration. At 24–48 hours post inoculation (hpi), green fluorescence was distributed on the chloroplast outer membrane (Fig. 4A and B). Additionally, expression of CaOMP24-GFP induced stromules on the chloroplasts (Fig. 4B). The green fluorescence from CaOMP24-GFP merged with the red fluorescence from the transit peptide of ferredoxin NADP(H) oxidoreductase (FNR-mCherry), a marker of stromules (Fig. 5). Notably, the chloroplasts progressively moved towards the nucleus when CaOMP24-GFP was expressed, finally being clustered around the nucleus (Fig. 4B).

CaOMP24 is a predicted to be a membrane protein. A transmembrane domain (TMD) exists at aa position 107–126, overlapping with the conserved motif near the C-terminal (Fig. 4A; Fig. S8A, see online supplementary material) (https://services.healthtech.dtu.dk/service.php?TMHMM-2.0). When the TMD was deleted, GFP-fused CaOMP24^ΔTMD^ mutant (CaOMP24^ΔTMD^-GFP) localized to the cell periphery and nuclear envelope but did not induce perinuclear chloroplast clustering (PCC) (Fig. 4A–B). When only the TMD was fused with GFP, the fluorescence was localized to the chloroplast outer membrane as well as the cell periphery and nuclear envelope but did not induce either stromules or PCC (Fig. 4B). In the TMD, the phenylalanine (F) at residue 118 of CaOMP24 is a hydrophobic amino acid. If it was substituted with the hydrophilic amino acid, Lysine (K), the TMD is predicted to disappear (Fig. S8B, see online supplementary material). As expected, CaOMP24^F118K^-GFP localized to the cell periphery and nuclear envelope but did not induce stromules or PCC (Fig. 4B). Moreover, expression of CaOMP24 mutants (CaOMP24^ΔTMD^, CaOMP24^F118K^, CaOMP24^TMD^) resulted in significantly lower stromule induction compared to CaOMP24 expression (Fig. 5). The expression of CaOMP24 and its mutants were confirmed using anti-GFP antibody (Fig. S8C, see online supplementary material). NbOMP24.1 has the same characteristics as CaOMP24. NbOMP24.1 was also localized to the chloroplast outer membrane and induced chloroplast clustering around the nucleus, while expression of mutants with deletion of the TMD (NbOMP24.1^ΔTMD^) or substitution of phenylalanine for lysine (NbOMP24.1^F109K^) led to localization to the plasma membrane and did not cause chloroplast clustering (Fig. S9, see online supplementary material). Additionally, PMMoV infection induced stromules and PCC (Fig. S10, see online supplementary material). Taken together, these results demonstrate that the TMD, and the phenylalanine (F) residue in the TMD, are vital for OMP24 chloroplast localization and induction of stromules and PCC.

### ROS accumulated in cells expressing CaOMP24 but not CaOMP24^F118K^

**Figure 6:**
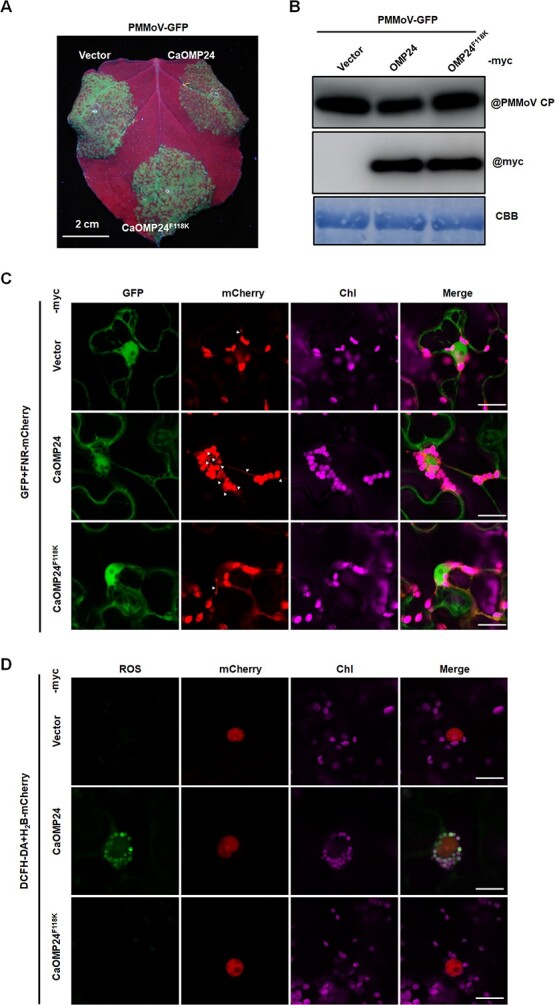
The phenylalanine (F) residue in the TMD of CaOMP24 is critical for its resistance to PMMoV. **A** GFP fluorescence in the region expressing CaOMP24 or CaOMP24^F118K^ inoculated with PMMoV-GFP. Photos were taken under UV light at 3.5 dpi. **B** Western blot detection of the expression of CaOMP24 and the accumulation of PMMoV CP. **C** Phenylalanine (F) residue in TMD of CaOMP24 is necessary for its induction of stromule formation. CaOMP24-myc and CaOMP24^F118K^-myc were independently co-expressed with GFP and FNR-mCherry via agroinfiltration; chloroplast clustering and stromule formation were examined at 48 hpi under the confocal microscope. 00-myc was used as a control. Scale bars, 20 μm. Triangles point to the stromules. **D** Total cellular ROS accumulation was measured by DCFH-DA staining. 48 hours after agro-infiltration of CaOMP24 and CaOMP24^F118K^-myc, *N. benthamiana* leaf discs were vacuum infiltrated with DCFH-DA and examined under the confocal microscope with excitation light of 488 nm; Scale bars, 20 μm.

Increasing evidence indicates that perinuclear chloroplast clustering (PCC) plays a fundamental defense role during plant-pathogen interactions [13, 14, 16, 38]. We therefore supposed that CaOMP24 might play its defensive role by inducing PCC. To investigate this, we first tested whether the CaOMP24 mutant that did not induce PCC was still able to defend against PMMoV. CaOMP24-Myc or CaOMP24^F118K^-Myc were co-infiltrated with PMMoV-GFP into *N. benthamiana* leaves by agroinfiltration. At 4 dpi, the intensity of fluorescence in zones expressing CaOMP24-Myc was less than that in control zones expressing 00-Myc (empty vector), confirming that CaOMP24 expression decreased PMMoV-GFP infection (Fig. 6A). However, the intensity of fluorescence in zones expressing CaOMP24^F118K^-Myc was similar to that in the control (Fig. 6A). Consistently, PMMoV CP accumulated at lower levels in the zones expressing CaOMP24 than in those expressing CaOMP24^F118K^-Myc or the control (Fig. 6B). These results suggest that the induction of PCC is essential for the defense function of CaOMP24.

It is hypothesized that PCC helps to transfer the retrograde signal ROS into the nucleus to regulate immune responses via stromules [26]. Consistent with the results obtained with CaOMP24-GFP (Figs 4B and 5), the frequency of stromule formation connecting chloroplasts and nuclei in CaOMP24-Myc was higher than that of the control and CaOMP24^F118K^-Myc (Fig. 6C). Furthermore, we monitored the total cellular ROS accumulation in cells expressing *CaOMP24* and its mutants using 2′,7′-dichlorodihydrofluorescein diacetate (DCFH-DA) staining. The accumulation of ROS in cells expressing *CaOMP24* was dramatically increased compared to the control or cells expressing CaOMP24^F118K^ (Fig. 6D). Consistent with the DCFH-DA staining results, H_2_O_2_ content in leaves expressing *CaOMP24* was significantly higher than in leaves expressing 00-myc, while this increase was compromised upon PMMoV infection (Fig. S11, see online supplementary material). Moreover, stromules and PCC were also significantly induced in *NbOMP24.1*-transgenic plants (OE#2 and OE#9), accompanied by an accumulation of ROS (Fig. S12A–C, see online supplementary material). As expected, the expression of PR1 and PR2, the resistant genes in ROS-mediated defense, was upregulated significantly in *NbOMP24.1*-transgenic plants (Fig. S12D, see online supplementary material). These results suggest that CaOMP24 may resist viral infection by the PCC-associated ROS-mediated defense pathway.

ROS are important signals mediating general resistance against pathogens [9, 51]. We here also investigated whether NbOMP24 functions in defense against two other viruses, potato virus X (PVX, genus *Potexvirus*) and turnip mosaic virus (TuMV, genus *Potyvirus*). Both viruses caused more severe disease symptoms and viral CP accumulation was enhanced in plants where *NbOMP24s* were silenced (Fig. S13, see online supplementary material), while on NbOMP24.1 transgenic plants the severity of viral disease symptoms and CP accumulation were decreased significantly (Fig. S14, see online supplementary material). These results indicated that NbOMP24 confers general resistance against different viruses.

### PMMoV CP impairs CaOMP24-induced PCC and accumulation of ROS by interfering with the CaOMP24 self-interaction

**Figure 7:**
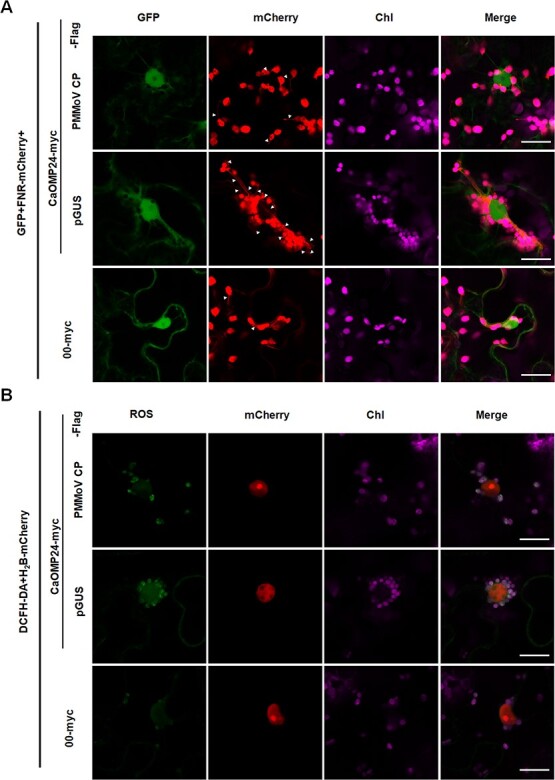
PMMoV CP impairs CaOMP24-induced stromules, PCC, and ROS production. **A** CaOMP24-induced perinuclear clustering of chloroplasts and stromule formation were impaired by PMMoV CP. The combination of CaOMP24-myc, GFP and FNR-mCherry was co-expressed with PMMoV CP-Flag or pGUS-Flag via agro-infiltration. Fluorescence signals were detected by confocal microscopy at 48 hpi. Scale bars, 20 μm. **B** DCHF-DA staining revealed that ROS accumulation induced by CaOMP24-myc was reduced in the presence of PMMoV CP. Scale bars, 20 μm.

Because PMMoV CP interacted with CaOMP24, we next investigated the effect of PMMoV CP on the ability of CaOMP24 to induce PCC and ROS accumulation. Confocal analysis at 48 hpi revealed that the extent of PCC and stromule induction was less in leaves co-expressing CaOMP24-myc with PMMoV CP-Flag than in the control (CaOMP24-myc + pGUS-Flag) (Fig. 7A). Meanwhile, cellular ROS accumulation induced by CaOMP24 was obviously decreased in the presence of PMMoV CP (Fig. 7B). In addition, the mRNA and protein expression levels of *CaOMP24* were not affected by co-expression with PMMoV CP (Fig. S15, see online supplementary material). These results indicate that PMMoV CP inhibits the antiviral function of CaOMP24.

**Figure 8:**
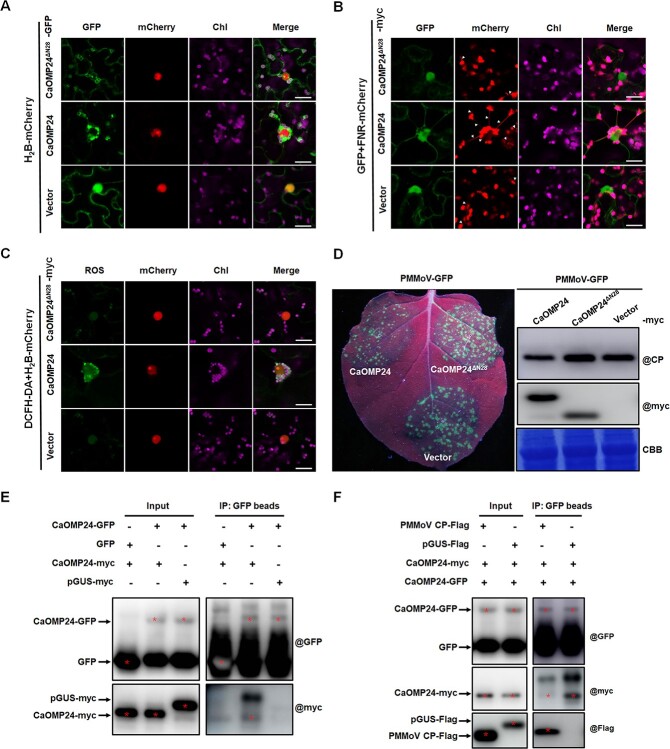
PMMoV CP interferes with the self-interaction of CaOMP24. **A** Subcellular localization of CaOMP24^ΔN28^-GFP with H2B-mCherry in *N. benthamiana* leaf 48 hours after agroinfiltration examined under the confocal microscope. Scale bars, 20 μm. **B** Co-expression of CaOMP24^ΔN28^-GFP with FNR-mCherry. The frequency of stromule formation was lower when CaOMP24^ΔN28^ was expressed compared to that of CaOMP24. Scale bars, 20 μm. Triangle, stromule. **C** Cellular ROS accumulation in cells expressing CaOMP24^ΔN28^-myc was measured by DCFH-DA staining. Scale bars, 20 μm. **D** CaOMP24^ΔN28^ did not confer resistance to PMMoV. GFP fluorescence and PMMoV CP accumulation in the regions expressing either CaOMP24 or CaOMP24^ΔN28^ with PMMoV-GFP at 4 dpi. **E** Co-IP assay confirmed the self-interaction of CaOMP24. The red asterisks indicate the expected band sizes. **F** Competitive Co-IP assays demonstrating that the self-interaction of CaOMP24 was disrupted by PMMoV CP. The combination of CaOMP24-GFP + CaOMP24-myc was co-expressed with PMMoV CP-Flag or pGUS-Flag in *N. benthamiana* leaves. Total protein was extracted with GTEN buffer and inoculated with anti-GFP Magnetic Beads, then the accumulation of CaOMP24-myc was detected by Western blot in coprecipitated protein. The red asterisks indicate the expected band sizes.

Next, we wanted to know how CP impaired CaOMP24-induced PCC and accumulation of ROS. Because we had determined (above) that the TMD of CaOMP24 was important for its localization and induction of PCC and that the N terminal 1-28aa of CaOMP24 was the key domain for its interaction with PMMoV CP, we tested whether the N terminus 1-28aa of CaOMP24 was also essential for inducing stromules, PCC, and ROS accumulation. GFP-fused CaOMP24^ΔN28^ localized at chloroplast outer membranes but did not induce stromules, PCC, or ROS accumulation (Fig. 8A–C). In addition, overexpression of CaOMP24^ΔN28^ did not confer resistance to PMMoV (Fig. 8D). These results indicate that the N terminal 1-28aa of CaOMP24 are also essential for CaOMP24-induced stromules, PCC, and defense.

Several chloroplast OMPs including Toc33 and Toc159 have been reported to require self-interaction to perform their functions [7, 57]. To analyse whether CaOMP24 functioned by interacting with itself, we performed Y2H, Co-IP, and LCI experiments to detect its self-interaction. CaOMP24 indeed interacted with itself (Fig. 8E; Fig. S16, see online supplementary material) and the key region for its self-interaction was in the N terminal 1–80 aa. Although CaOMP24 interacted with all three truncated mutants, CaOMP24 [1–28], CaOMP24 [29–49], and CaOMP24 [50–80], CaOMP24 had a higher infinity to CaOMP24 [1–28] than the other two (Fig. S16B, see online supplementary material). These results show that the key region of CaOMP24 for self-interaction is located at the N-terminal 1-80aa, and especially the N-terminal 1-28aa.

Because the N-terminal 1–28 aa of CaOMP24 is important both for self-interaction and for that with PMMoV CP, we analysed whether PMMoV CP interfered with the self-interaction of CaOMP24 by competitively binding the N-terminal 1–28 aa of CaOMP24. In competitive Co-IP experiments, PMMoV CP-Flag was co-infiltrated with the combination of CaOMP24-GFP and CaOMP24-myc. pGUS-Flag was used as the control. The CaOMP24-myc immunocaptured by GFP magnetic beads was significantly reduced by expression of PMMoV CP-Flag (Fig. 8F). Consistently, in the LCI experiment, the signal generated by CaOMP24 self-interaction was weakened in the presence of PMMoV CP (Fig. S17, see online supplementary material). These results revealed that PMMoV CP impaired CaOMP24-induced PCC by interfering with the self-interaction of CaOMP24.

## Discussion

Numerous studies have demonstrated the important role of chloroplasts in plant-virus interactions [10, 60]. Many chloroplast-related proteins have also been identified to be involved in viral replication, movement, pathogenicity, and plant antiviral immunity. Although chloroplast membranes can be targeted and rearranged by several viruses to serve as viral replication sites [12, 35], the function of proteins located at the chloroplast membrane in the chloroplast-virus interactions is not well studied. Here, we identified OMP24 that played defense roles against viruses by inducing stromules, PCC, and ROS accumulation, which broadens our knowledge of the function of OMPs in plants.

Chloroplasts possess a double membrane system, consisting of outer and inner envelope membranes. Proteins targeted to the outer envelope membrane are classified into several groups, signal-anchored (SA), tail-anchored (TA), β-barrel, and other proteins [19]. SA and TA proteins contain a single α-helix transmembrane domain located at their N- and C-terminals, respectively [19, 31, 37]. The moderately hydrophobic TMD is important for SA and TA proteins targeting to the outer membrane [32]. OMP24 has a single transmembrane domain (Fig. 4; Figs S7 and S8, see online supplementary material) but, unlike SA and TA proteins, this TMD is located in the middle of the sequence towards the C-terminal (Figs S7 and S8, see online supplementary material). Similar to SA and TA proteins, the hydrophobic TMD is also essential for OMP24 targeting to the outer membrane (Fig. 5; Fig. S9, see online supplementary material). The TMD of OMP24 protein is highly conserved in *C. annuum, N. benthamiana, A. thaliana*, and *S. oleracea* (Fig. S1, see online supplementary material), indicating that the TMD may play important roles in the functions of plant OMP24 proteins. Besides the TMD, SA and TA proteins need other structural features to anchor in the membrane, including a C-terminal positively charged region (CPR) in SA and a C-terminal sequence (CTS) in TA [40, 46]. In contrast, it seems as if the TMD of OMP24 localizes at the membrane by itself, without the assistance of other segments (Fig. 4; Fig. S8, see online supplementary material). These results suggest that OMP24 may differ from SA and TA proteins.

**Figure 9:**
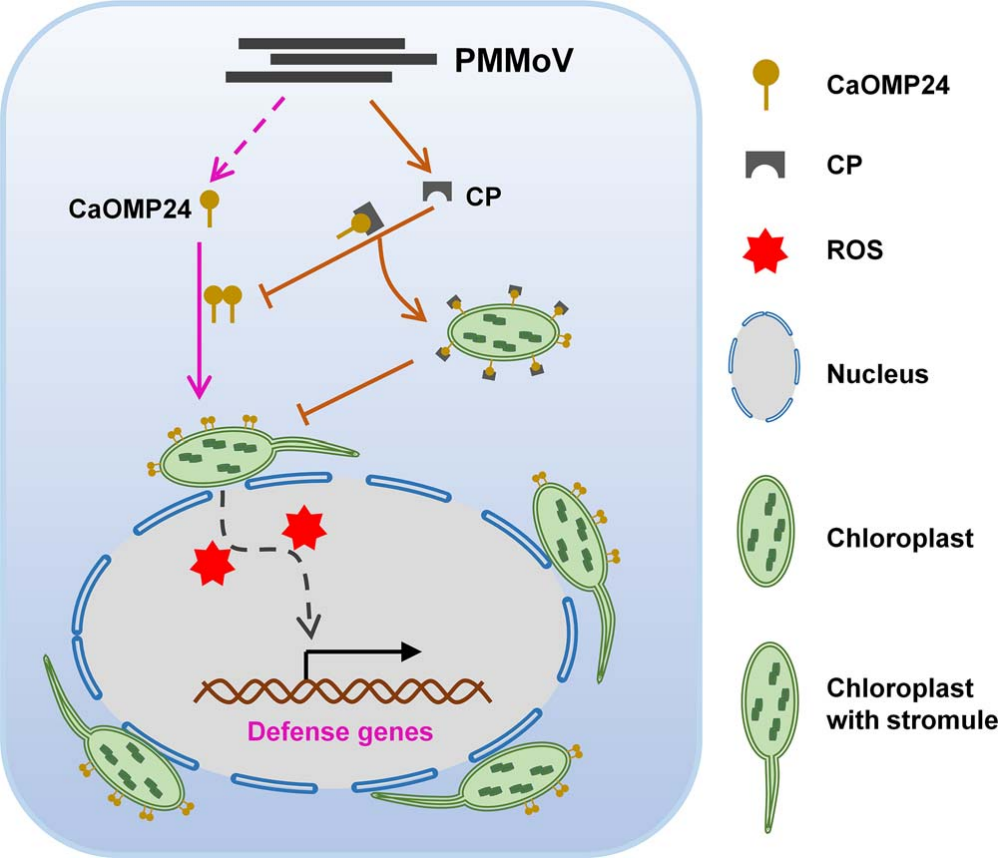
A proposed model for the antiviral function of CaOMP24 in PMMoV infection. Upon perception of PMMoV infection, CaOMP24 is rapidly upregulated to induce stromules, perinuclear chloroplast clustering, and accumulation of reactive oxygen species (ROS). These consequences are required for the self-interaction of CaOMP24 and confer resistance to PMMoV. To counter this defense response, PMMoV CP interacts with CaOMP24 and interferes with the self-interaction of CaOMP24, thus suppressing the antiviral defense mediated by CaOMP24 to facilitate PMMoV infection.

The correlation between perinuclear clustering of plastids and immune responses is conserved in animals and plants. In mammalian cells, perinuclear mitochondrial clustering results in ROS accumulation in the nucleus and is important for hypoxia-induced transcriptional regulation and heat shock response [2, 3]. In addition, perinuclear mitochondrial clustering has a role in the regulation of cellular calcium transport [47]. Interestingly, overexpression of mitochondrial outer-membrane proteins, such as EXD2 and hFis1, results in mitochondrial perinuclear clustering [20, 30]. In plants, PCC is a general response of plants under biotic and abiotic stresses [16, 17, 42]. In *N. benthamiana*, expression of the *Xanthomonas* effector XANTHOMONAS OUTER PROTEIN Q (XopQ) or TMV p50 activates ETI immunity accompanied by chloroplast relocation around the nucleus [49]. Stromules are associated with PCC and are thought to guide chloroplasts to the nucleus and to transfer the retrograde signaling such as ROS from chloroplasts to the nucleus to regulate resistance gene expression [13, 16, 26, 27]. In our recent work, we identified that upregulated NbNdhM contributed to plant defense against TuMV by inducing PCC and stromules [58]. We here provide evidence demonstrating that OMP24 induces stromules, PCC, and accumulated ROS, and contributes to plant defense. Both results further support the conclusion that the PCC-associated retrograde signaling pathway plays an essential role in plant defense against viruses.

Viruses have numerous strategies to regulate or evade plant immune responses for their own successful infection [11, 54]. Emerging evidence suggests that the chloroplast is a central player in perception of pathogen invasion and innate immunity by functioning as a source of defense signals, including phytohormones, ROS, and calcium (Ca^2+^), and that many pathogens have strategies to attenuate chloroplast-mediated immunity [10, 36, 42, 60]. For instance, re-localization of tomato yellow leaf curl virus (TYLCV) C4 protein from the plasma membrane to the chloroplast aids viral infection as the C4 protein can suppress chloroplast-specific defense mechanisms, specifically the biosynthesis of salicylic acid [45]. TuMV Vpg impairs the antiviral PCC induced by NbNdhM [58]. TuMV P1 interacts with chloroplast protein cpSRP54 to suppress JA-mediated resistance [33]. Barley stripe mosaic virus γb protein disrupts chloroplast antioxidant defenses by interfering with interactions between NTRC and 2-Cys Prx [53]. We here showed that PMMoV CP suppresses the antiviral defense mediated by OMP24 by interfering with the self-interaction of OMP24 (Fig. 9), which provides evidence that the viral structural protein CP also plays anti-defense roles by this mechanism. Meanwhile, CPs of TuMV and PVX could not interact with OMP24 (Fig. S18, see online supplementary material). It is possible that other viral factors of both viruses might play anti-defense roles.

## Materials and methods

### Plant growth conditions and virus inoculation

*N. benthamiana* and *C. annuum* L. cultivar Haonong11 plants were grown in growth chambers with 14-h-light/10-h-dark photoperiod at 24–26°C.

Infectivity assays in *N. benthamiana* were performed using PMMoV-GFP, PMMoV, TuMV-GFP, or PVX-GFP infectious clones. Virus symptoms were monitored daily and the GFP fluorescence resulting from viral infection was observed under UV light. For PMMoV inoculation in pepper, sap of *N. benthamiana* leaves infected with PMMoV was inoculated onto leaves of pepper seedlings by mechanical inoculation.

### Plasmid construction

The full-length CDs of OMP24 were amplified by PCR using KOD FX DNA Polymerase (Toyobo, Osaka, Japen) from cDNA of *N. benthamiana* and *C. annuum*. The fragments of NbOMP24^ΔTMD^, NbOMP24^F109K^, CaOMP24^ΔTMD^, and CaOMP24^F118K^ were obtained by Overlapping PCR.

PMMoV CP, CaOMP24, NbOMP24 and its mutants were introduced into different Ligation independent cloning (LIC) vectors: pCV-LIC-N-nYFP (C-terminal nYFP), pCV-LIC-N-cYFP (C-terminal cYFP), pCV-LIC-N-nLUC (C-terminal nLUC), pCV-LIC-C-Cluc (N-terminal cLUC), pCV-LIC-N-GFP (C-terminal GFP), pCV-LIC-N-mCherry (C-terminal mCherry), pCV-LIC-N-4 × Myc (C-terminal 4 × Myc), pCV-LIC-N-3 × Flag (C-terminal 3 × Flag) [8]. These LIC vectors for transient expression analysis were modified from a previously described pJG045 vector [59].

For the yeast two hybrid (Y2H) assays, PMMoV CP, NbOMP24, CaOMP24 and its mutants were inserted into the bait vector (pDHB1) or the prey vector (pPR3-N) using the *SfiI* restriction site, respectively.

A partial fragment of NbOMP24 (216 nt) and CaOMP24 (283 nt) was amplified and cloned into pTRV2, producing the vectors pTRV2:NbOMP24 and pTRV2:CaOMP24, respectively. To generate hairpin silencing construct targeting NbOMP24, the sequence of NbOMP24 used for VIGS was inserted into pFGC5941 in both sense and antisense orientations to construct the hpNbOMP24 vector. The hpGUS vector used for the control was as previously described [33].

All primers used for plasmid construction are listed in Table S1 (see online supplementary material).

### Agroinfiltration of *N. benthamiana* plants

For *Agrobacterium tumefaciens*-mediated transient expression, transient expression vectors were electroporated into *A. tumefaciens* strain GV3101. The cultures of *A. tumefaciens* were re-suspended in infiltration solution and were infiltrated onto the underside of leaves. The infiltrated leaves were sampled 24–72 hours post infiltration for the corresponding assays.

### Virus-induced gene silencing in *C. annuum* and *N. benthamiana*

To silence NbOMP24, pTRV2:NbOMP24 was co-expressed with pTRV1 in *N. benthamiana* plants by agroinfiltration. Empty vector (pTRV2:00) combined with pTRV1 served as the control. Plants silenced for 12 days were inoculated with PMMoV-GFP. For silencing of CaOMP4 in pepper, pTRV2:CaOMP24 and pTRV1 were mixed at a 1:1 ratio and agro-infiltrated onto 2-leaf stage pepper leaves. Pepper plants silenced for 18 days were used for further analysis.

### Split ubiquitin yeast two-hybrid assays

The yeast two-hybrid assays based on the split-ubiquitin technique were performed using the DUAL hunter starter kit (Clonetech, Mountain view, California, USA). PMMoV CP, NbOMP24, CaOMP24 and its mutants were inserted into the pDHB1 or pPR3-N vectors, followed by transformation into the NMY51 yeast strains by a lithium acetate method. The transformed yeast cells were then plated on SD/−Leu/−Trp to verify successful co-transformation and then transferred onto SD/−Leu/−Trp/-His/−Ade dropout medium to assess the interaction of the target proteins.

### Luciferase complementation imaging assays

Split-luciferase complementation assays were performed as described previously [52].

### Western blot and co-immunoprecipitation (co-IP) assays

For western blot, total proteins were extracted from pepper or *N. benthamiana* leaves with lysis buffer. The proteins were separated by SDS–PAGE, and subsequently transferred onto Immobilon®-P PVDF Membrane (Merck, Co. Cork, Ireland) using the wet transfer method. The membranes were probed with primary antibody (Myc, GFP, Flag, PMMoV CP, TuMV CP, and PVX CP) and HRP-conjugated (anti-Mouse or anti-Rabbit) secondary antibodies (TransGen Biotech, Beijing, China) followed by ECL detection using Immobilon Western HRP Substrate (Millipore, Darmstadt, Germany). The Rubisco large subunit protein (RbcL) stained with Coomassie Brilliant Blue R-250 Dye was used as a loading control.

The co-IP assays were performed as described previously [34]. The proteins to be tested were transiently expressed by Agrobacterium infiltration in *N. benthamiana* plants. Total proteins from infiltrated *N. benthamiana* leaf tissue were extracted and incubated with GFP-Trap® beads (ChromoTek, Planegg-Martinsried, Germany). The precipitates were washed at least three times with GTEN buffer containing 0.1% NP-40 and analysed by Western blotting using anti-GFP, anti-Myc, or anti-Flag antibodies.

### qRT-PCR analysis

Total RNA was isolated from pepper or *N. benthamiana* leaves using Trizol reagent (Invitrogen, Carlsbad, California, US). The first-strand cDNA was synthesized using the ReverTra Ace™ qPCR RT Master Mix with gDNA Remover kit (Toyobo, Osaka, Japen). qRT-PCR was conducted on the LightCycler®480 Real-Time PCR System (Roche, Mannheim, Germany) using SYBR Green qPCR Master Mix (Vazyme, Nanjing, China) in accordance with the manufacturer’s instructions. The results were analysed by the ΔΔCT method [43]. *C. annuum β-Tublin* or *Nbactin* was used as the reference gene. At least three biological replicate samples were used. The primers used for qRT-PCR are listed in Table S1 (see online supplementary material).

### Laser confocal microscopy assays

Subcellular localization and bimolecular fluorescence complementation (BiFC) assays were performed as described previously [58]. The agro-infiltrated *N. benthamiana* leaves were observed at 24 to 48 hpi using a Nikon A1 confocal microscope.

### Generation and identification of NbOMP24 transgenic *N. benthamiana* plants

The NbOMP24 transgenic *N. benthamiana* plants were generated by leaf disk transformation with Agrobacterium containing pCV-NbOMP24.1-Myc [44]. Western blot with anti-myc antibody (TransGen Biotech, Beijing, China) was performed to screen the transgenic plants.

### Detection of cellular ROS by DCFH-DA staining

Reactive oxygen species (ROS) accumulation measured by DCFH-DA staining was performed as described previously [50]. A 100 mM stock solution of DCFH-DA (MCE, Shanghai, China) was prepared by dissolving 100 mg in 2.0522 mL DMSO. Leaf discs from agro-infiltrated and transgenic plants overexpressing NbOMP24 were stained with 50 μM DCFH-DA in 10 mM Tris–HCl (pH 7.5) by vacuum-infiltration and inoculated in the darkness for 30 min. ROS accumulation was visualized as green fluorescence in a Nikon A1 confocal microscope with excitation at 488 nm and emission at 515–530 nm.

### Detection of H_2_O_2_ and superoxide

H_2_O_2_ and Superoxide were detected visually in leaves using 3,3’-diaminobenzidine (DAB, MCE, Shanghai, China) and Nitrotetrazolium blue chloride (NBT, MCE, Shanghai, China) staining, respectively [39]. H_2_O_2_ concentration was measured using a kit (Comin Suzhou, China) following the recommended protocol.

## Acknowledgements

We thank Prof. M.J. Adams, Minehead, UK for correcting the English of the manuscript. This work was financially supported by National Key R&D Program of China (2022YFD1401200), China Agriculture Research System of MOF and MARA (CARS-24-C-04), National Natural Science Foundation of China (32270294), Ningbo Major Special Projects of the Plan ‘Science and Technology Innovation 2025’ (2021Z106), the Postdoctoral Science Foundation of Anhui Province, China (2021B558) and K.C. Wong Magna Fund in Ningbo University.

## Author contributions

F.Y., K.H., R.Q., J.P. and J.C. designed the project. K.H., Ho.Z., D.Y., Hu.Z., Z.J., and Y.Z. performed experiments. K.H., F.Y., J.W., Y.L., G.W. and S.R. analysed data and reviewed the manuscript. K.H. and F.Y. wrote the paper.

## Data availability

All relevant data are available within the article and its supplementary materials.

## Conflict of interest

The authors declare that they have no competing interests.

## Supplementary data

Supplementary data is available at *Horticulture Research* online.

## Supplementary Material

Web_Material_uhad046
