# Supplementary material for: Pepper mild mottle virus coat protein interacts with pepper chloroplast outer envelope membrane protein OMP24 to inhibit antiviral immunity in plants
Source: Hortic Res. 2023 Mar 15;10(5):uhad046. doi: 10.1093/hr/uhad046 (PMC10170409; doi:10.1093/hr/uhad046)
Supplement: Web_Material_uhad046 [file web_material_uhad046.zip › Supplementary file.docx]

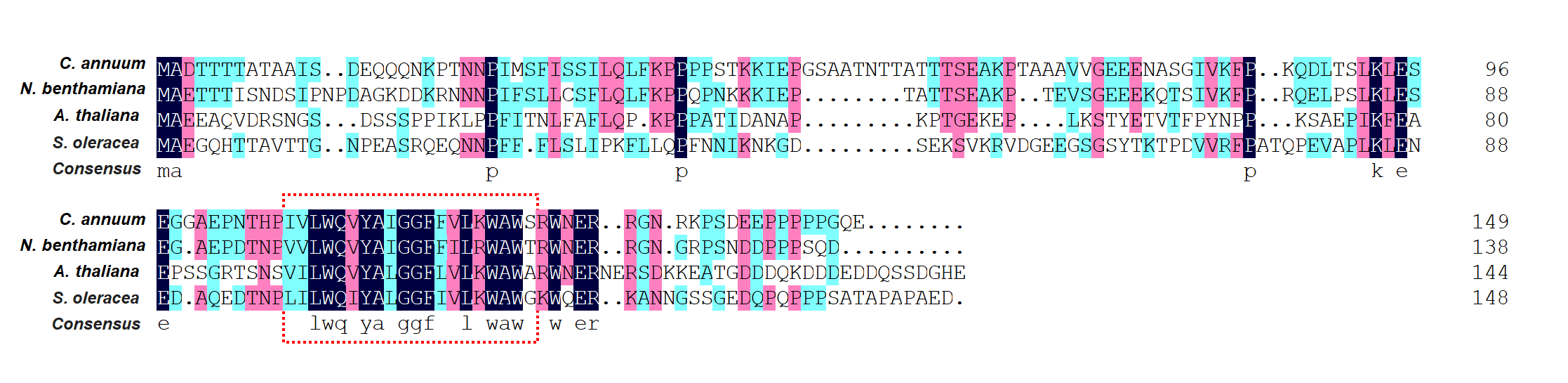


**Fig S1 Amino acid sequence alignment of OMP24s from *Capsicum annuum*, *N. benthamiana*, *Arabidopsis thaliana* and *Spinacia oleracea*.** Red dotted box shows the TMD.


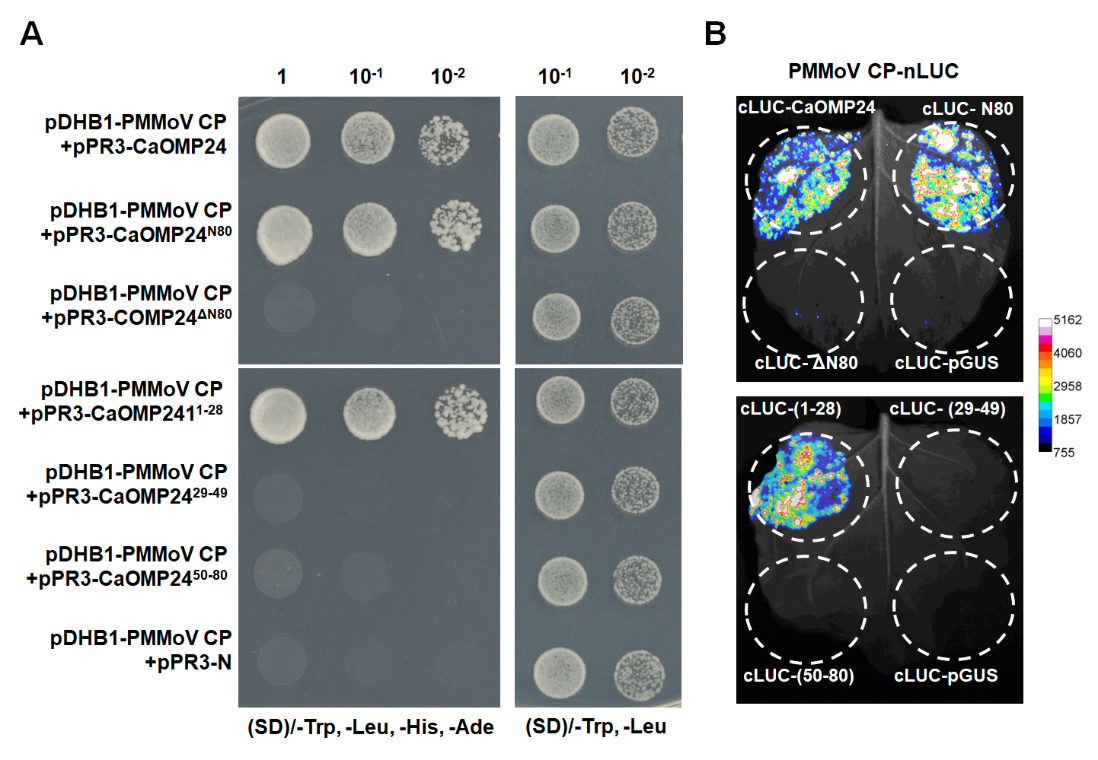


**Fig S2 Analysis of the region of CaOMP24 that interacts with PMMoV CP** (A) The five truncated mutants of CaOMP24 used are shown schematically. (B) Analysis of the key region of CaOMP24 in its interaction with PMMoV CP by Y2H assay. pPR3 constructs containing CaOMP24 and its mutants were independently transformed with pDHB1-PMMoV CP in NMY51 yeast strain. The construct pair pDHB1-PMMoV CP with pPR3-N was used as the negative control. (C) LCI assays analyzing the key region of CaOMP24 for its interaction with PMMoV CP.


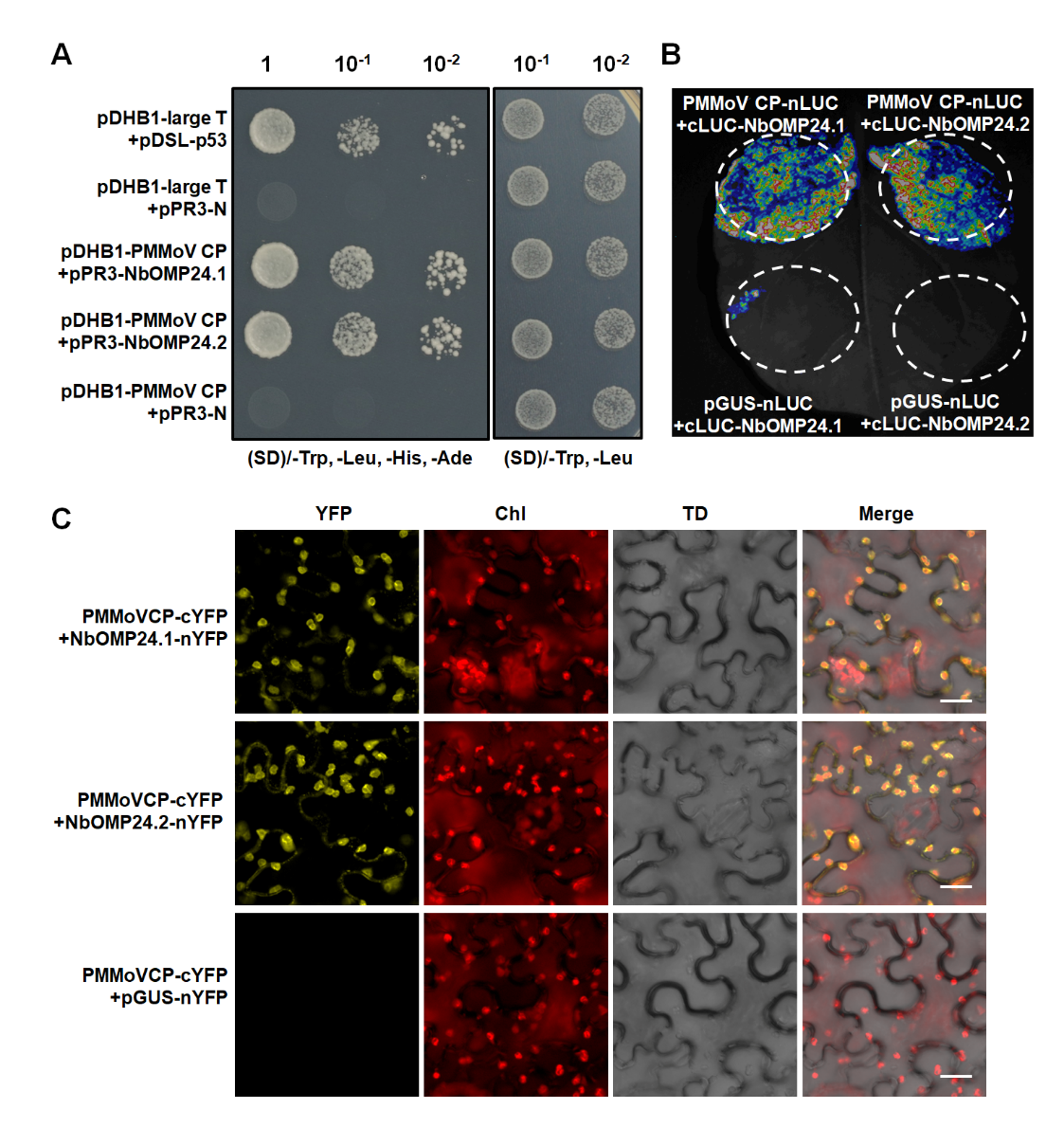


**Fig S3 PMMoV CP interacts with NbOMP24s.** Y2H (A), LCI (B) and BiFC (C) assays demonstrating the interaction between PMMoV CP and NbOMP24s. Scale bars = 20 μm.

**
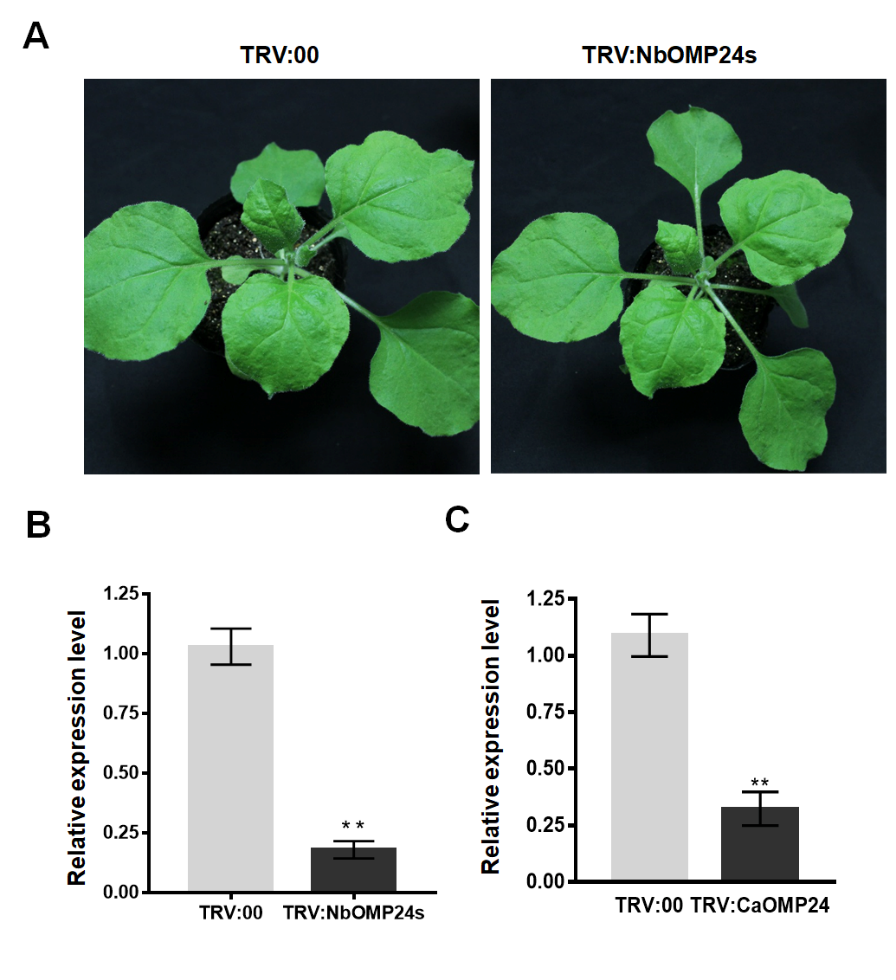
**

**Fig S4 Effect of TRV-induced *NbOMP24s* silencing on *N. benthamiana***. (A) Silencing of *NbOMP24s* caused no significant phenotypic changes compared to TRV:00 plants. Photographs were taken 12 days after TRV inoculation. (B) Detection of *NbOMP24s* mRNA levels in TRV:NbOMP24-infected plants by qRT-PCR. *Nbactin* was used as the reference gene. The mean expression values were analyzed using Student’s t-test (**, P ≤ 0.01). (C) qRT-PCR analysis of the silencing efficiency of CaOMP24. β-tubulin was used as reference gene. The mean expression values were analyzed using Student’s t-test (**, P ≤ 0.01).


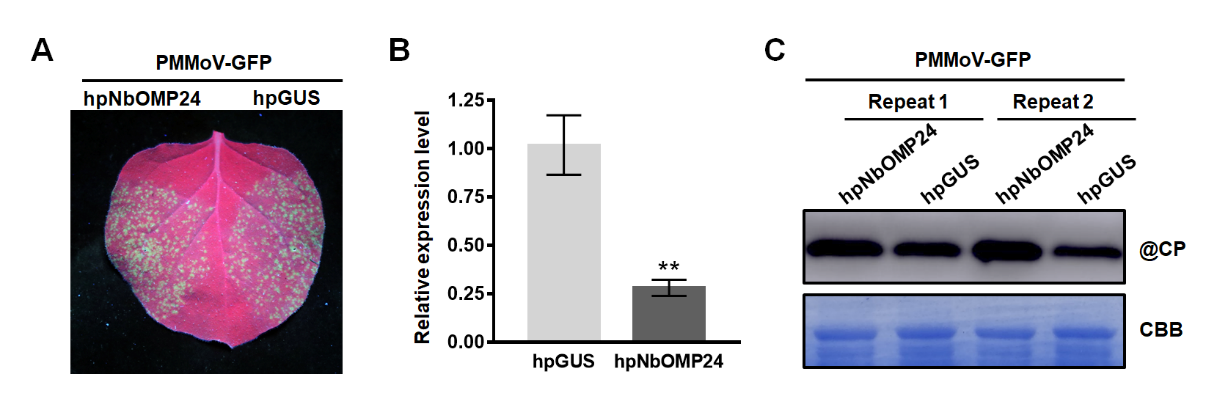


**Fig S5 Silencing of *NbOMP24s* by transient expression of the *NbOMP24* hpRNA (hpNbOMP24) construct facilitated infection by PMMoV.** (A) GFP fluorescence in leaves transiently expressimg hpNbOMP24 inoculated with PMMoV-GFP. Agrobacterium cultures harboring PMMoV-GFP were mixed with Agrobacterium harboring hpNbOMP24 (left) or hpGUS (right), and infiltrated into different parts of a single leaf of *N. benthamiana*. Photograph was taken 3.5 days under UV light after PMMoV-GFP inoculation. (B) qRT-PCR analysis of silencing efficiency of NbOMP24 by transient expression of hpNbOMP24. (C) Western blotting showing that the accumulation level of PMMoV CP in the region transiently expressing hpNbOMP24 was higher than the controls. Total protein was extracted from PMMoV-GFP-inoculated leaves at 3.5 dpi.


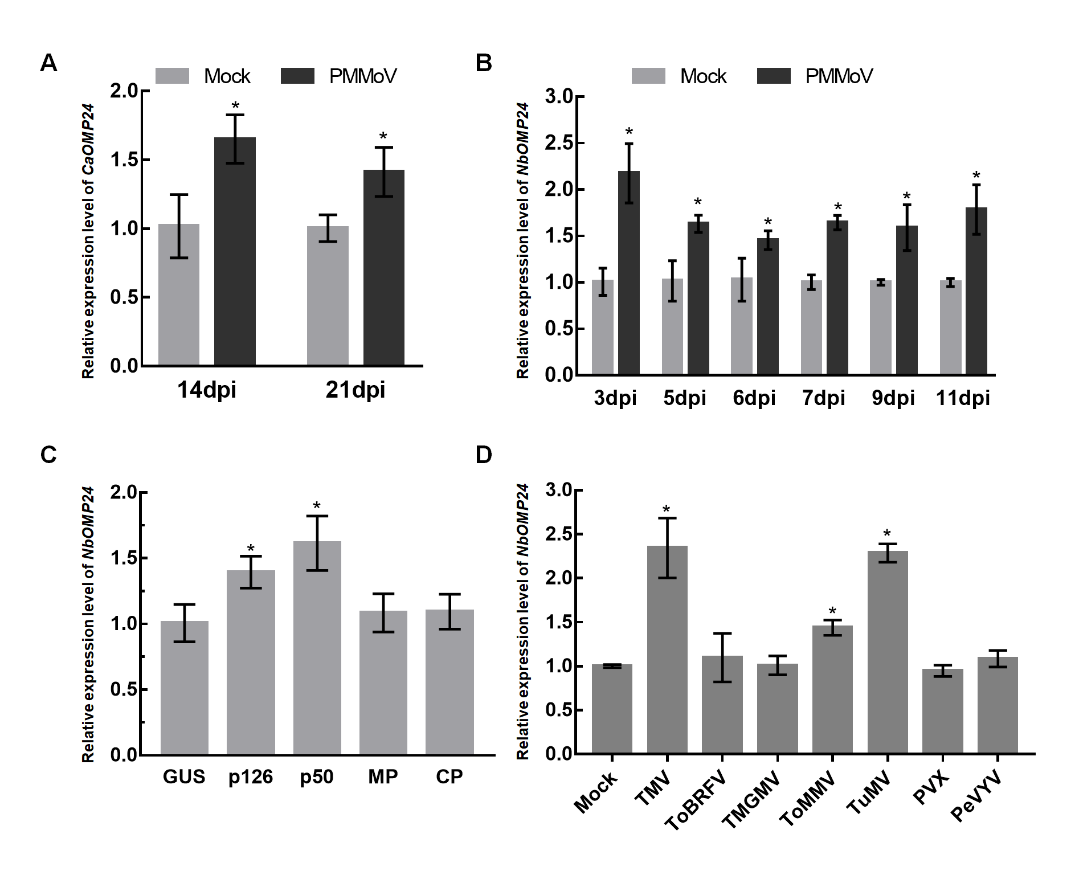


**Fig S6 qRT-PCR detection of the expression level of CaOMP24 and NbOMP24s.** (A) The expression pattern of CaOMP24 in PMMoV-infected pepper. Quantification of CaOMP24 mRNA levels in PMMoV infected peppers (cultivar No.11 Haonong) at 14 dpi and 21 dpi by qRT-PCR analysis. Mock-inoculated plants served as control. β-tubulin was used as the reference gene. The mean expression values were analyzed using Student’s t-test (*, P ≤ 0.05) (B) Time course of the expression level of NbOMP24s in N.benthamiana inoculated with PMMoV at 3, 5, 7, 9, 11 dpi. NbActin was used an internal reference gene. (*, P ≤ 0.05) (C) Relative expression levels of NbOMP24s in leaves expressing PMMoV p126, p50, MP or CP. (*, P ≤ 0.05) (D) qRT-PCR analysis of the expression level of NbOMP24s with seven other pepper-infecting viruses (tobacco mosaic virus (TMV), tomato brown rugose fruit virus (ToBRFV), tobacco mild green mosaic virus (TMGMV), tomato mottle mosaic virus (ToMMV), TuMV, PVX or pepper vein yellows virus (PeVYV)) infection in N. benthaniana. Virus-infected systemic leaves were collected at 10 dpi for analysis. (*, P ≤ 0.05).


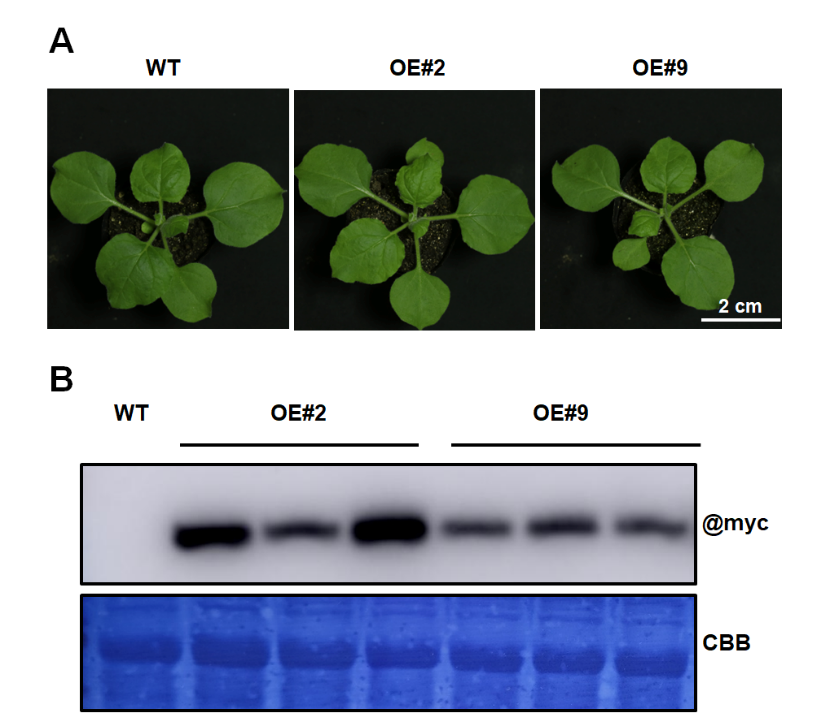


**Fig S7 The phenotype of NbOMP24.1-overexpression transgenic plants**. (A) The phenotype of WT and NbOMP24.1 transgenic plants (OE#2 and OE#9). Pictures were taken 18 days after transplanting. Scale bar, 5 cm. (B) The expression of NbOMP24.1-myc in lines OE#2 and OE#9 was confirmed by western blot using anti-myc antibody.


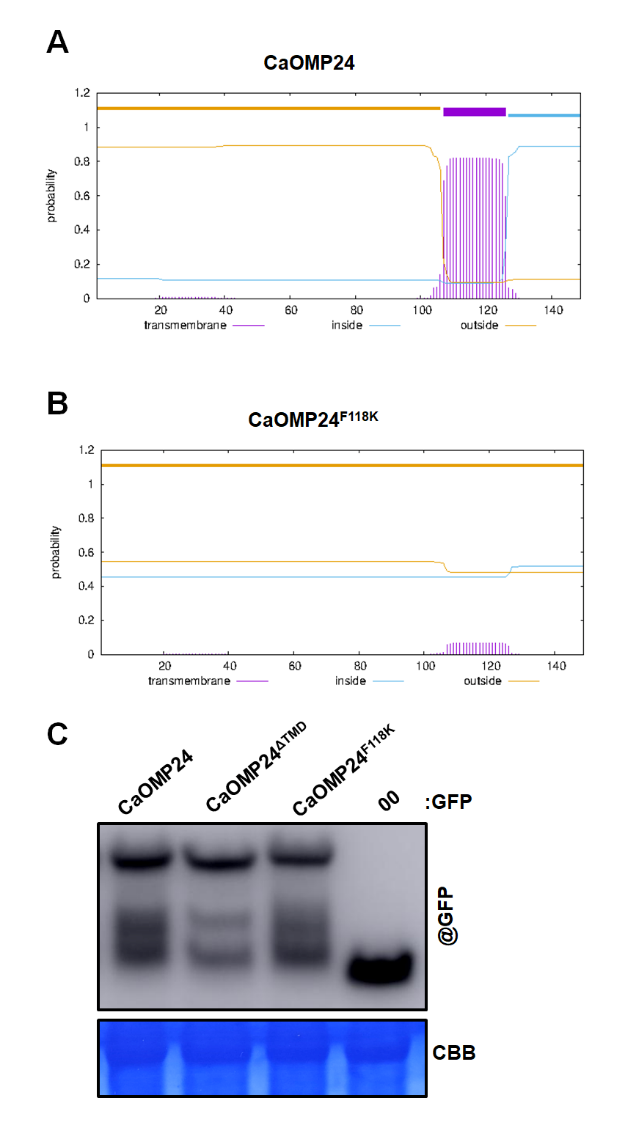


**Fig S8** **The expression of** **CaOMP24 and its mutants detected by western blotting.** (A, B) Predictions of Transmembrane Domain (TMD) in CaOMP24 and CaOMP24^F118K^ by TMHMM-2.0 (<https://services.healthtech.dtu.dk/service.php>? TMHMM-2.0). (C) Western blot confirming the expression of CaOMP24 and its mutants at 2 dpi in Fig 4.


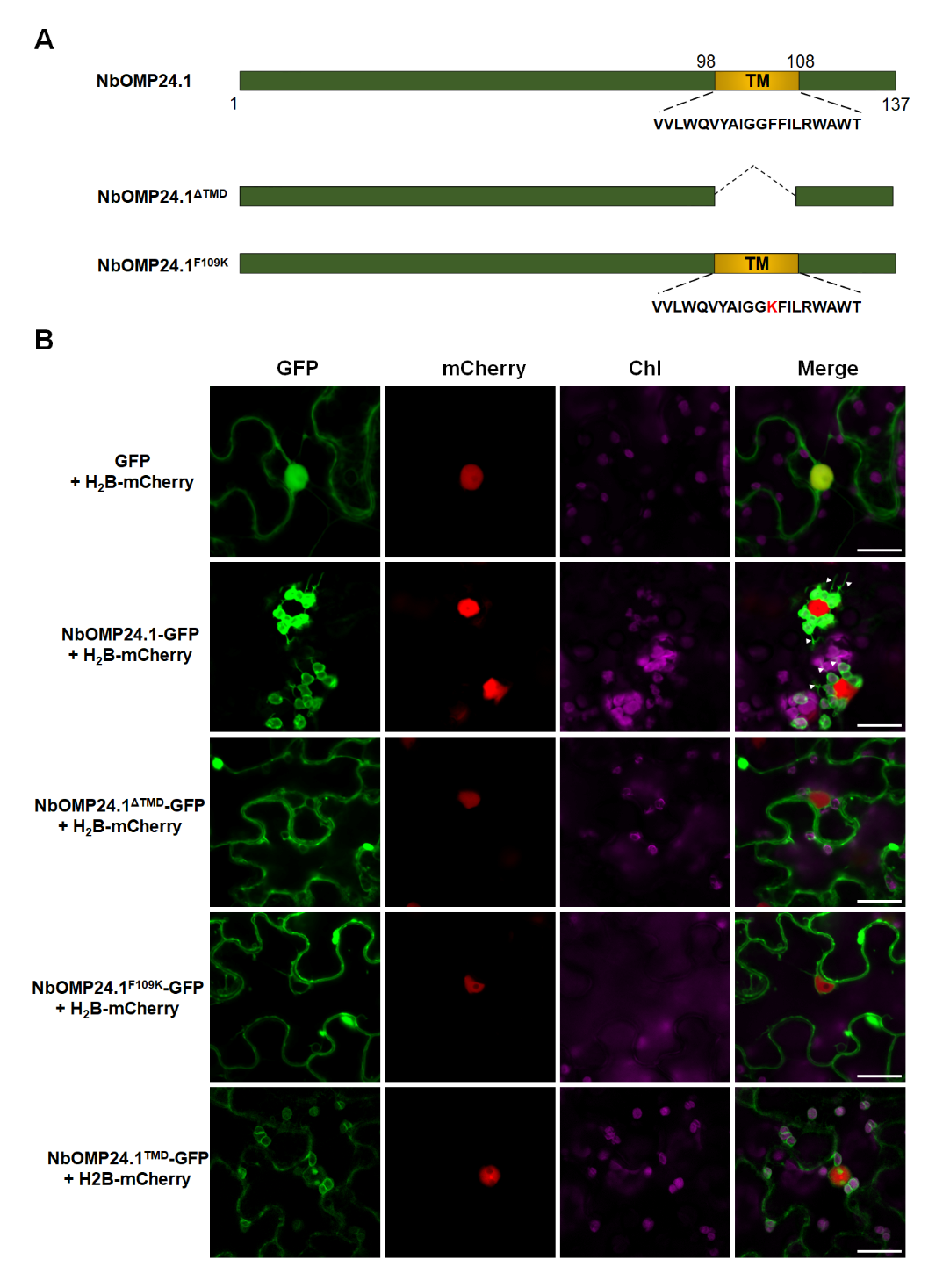


**Fig S9 The subcellular localization of** **NbOMP24.1 and its mutants.** (A) Schematic representation of NbOMP24.1 and its mutants. TMD, transmembrane domain; (B) NbOMP24.1-GFP, NbOMP24.1^ΔTMD^-GFP, NbOMP24.1^F109K^-GFP or NbOMP24.1^TMD^-GFP were co-expressed with H_2_B-mCherry via agro-infiltration. Fluorescence signals were examined by confocal microscopy at 48 hpi. GFP was used as a control. Scale bars, 20 μm.


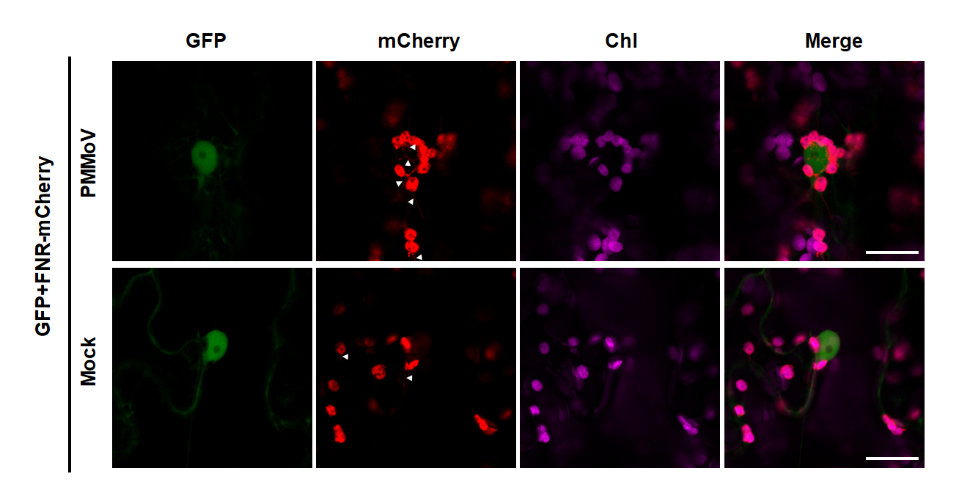


**Fig S10 Perinuclear chloroplast clustering and stromule formation were induced by PMMoV infection in *N. benthaniana* inoculated leaves.** pCB301-PMMoV were co-infiltrated with Agrobacterium harboring GFP and FNR-mCherry. pCB301-GUS was used as a control (Mock). Perinuclear chloroplast clustering and stromule formation were examined at 72 hpi under the confocal microscope. Scale bars, 20 μm.


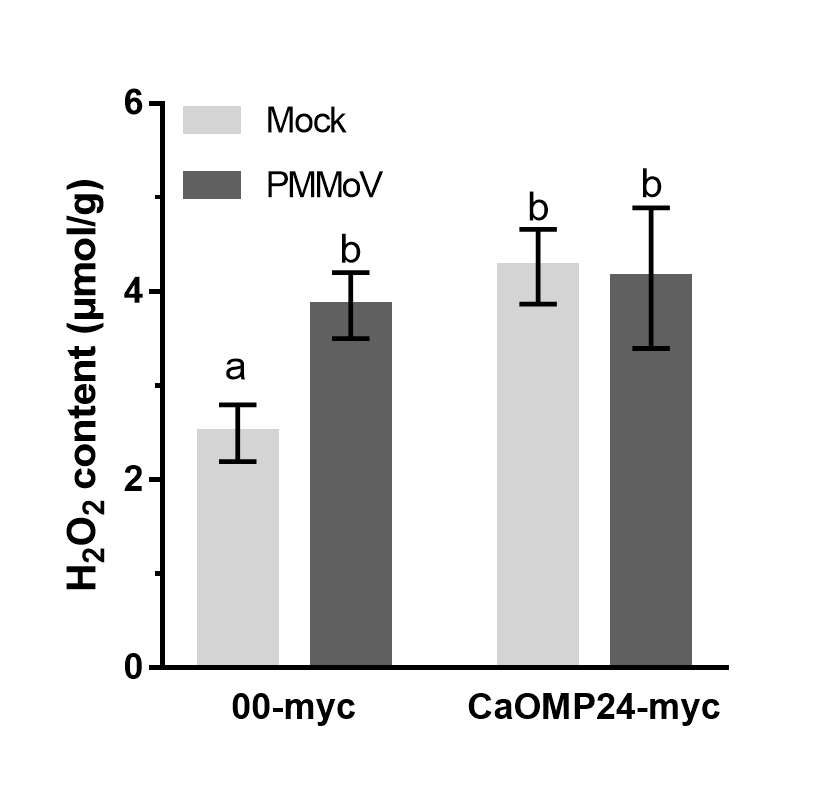


**Fig S11 H_2_O_2_ content in mock or PMMoV inoculated leaves was determined at 3.5 dpi.**


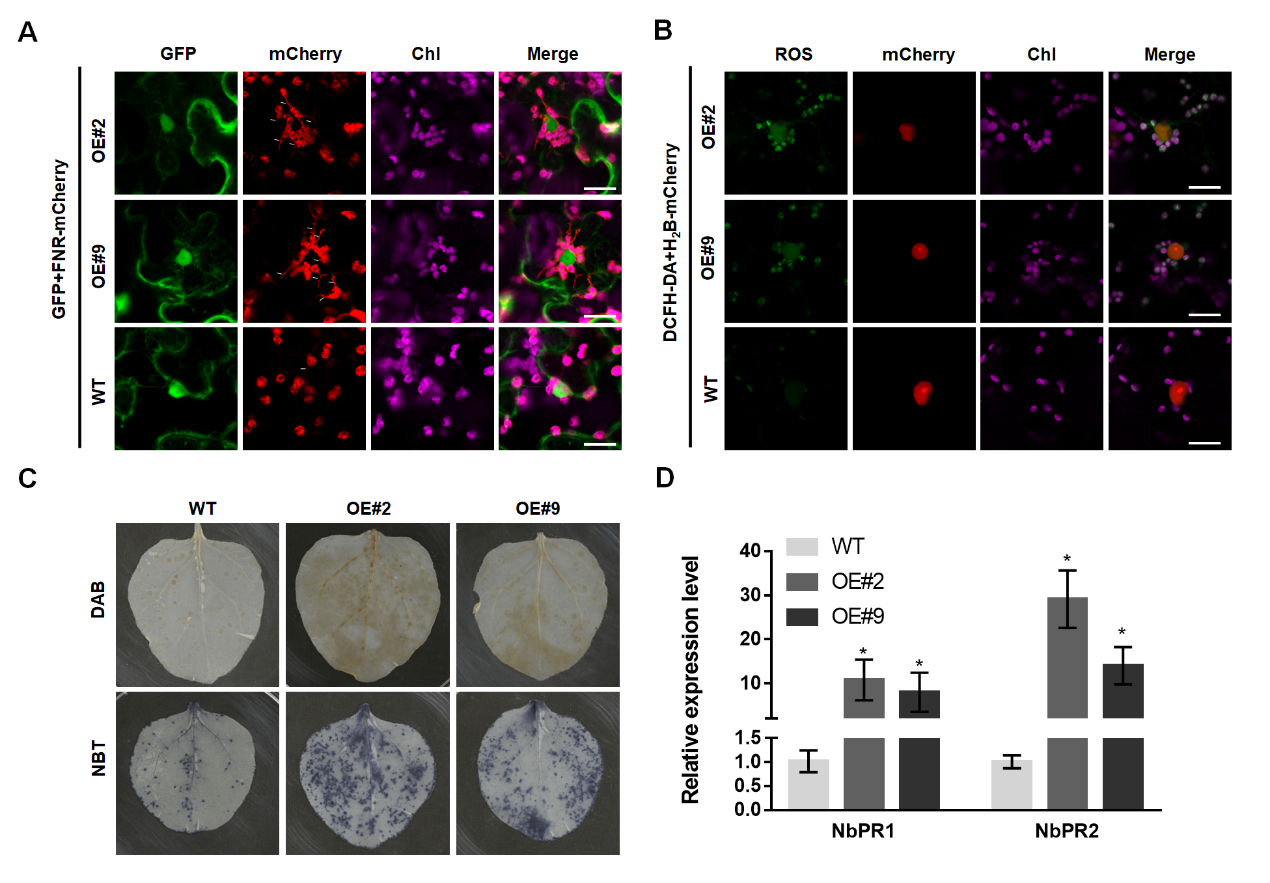


**Fig S12 Perinuclear chloroplast clustering, stromule formation and ROS accumulation were induced in** **transgenic plants overexpressing NbOMP24.1.** (A) NbOMP24.1-overexpression transgenic plants were infiltrated with Agrobacterium harboring GFP and FNR-mCherry. Perinuclear chloroplast clustering and stromule formation were examined at 48 hpi under the confocal microscope. Wild type *N. benthamiana* was used as a control. GFP: nuclear marker, FNR-mCherry: stromule marker. Scale bars, 20 μm. (B) Total cellular ROS accumulation in wild-type and transgenic plants overexpressing NbOMP24.1 were measured by DCFH-DA staining. Transgenic plants overexpressing NbOMP24.1 were stained with DCFH-DA via vacuum infiltration, ROS production as shown by the fluorescence excited at 488 nm. Scale bars, 20 μm. (C) DAB and NBT staining of transgenic plants overexpressing NbOMP24.1. (D) qRT-PCR analysis of the expression of PR1 and PR2 in transgenic plants overexpressing NbOMP24.1.


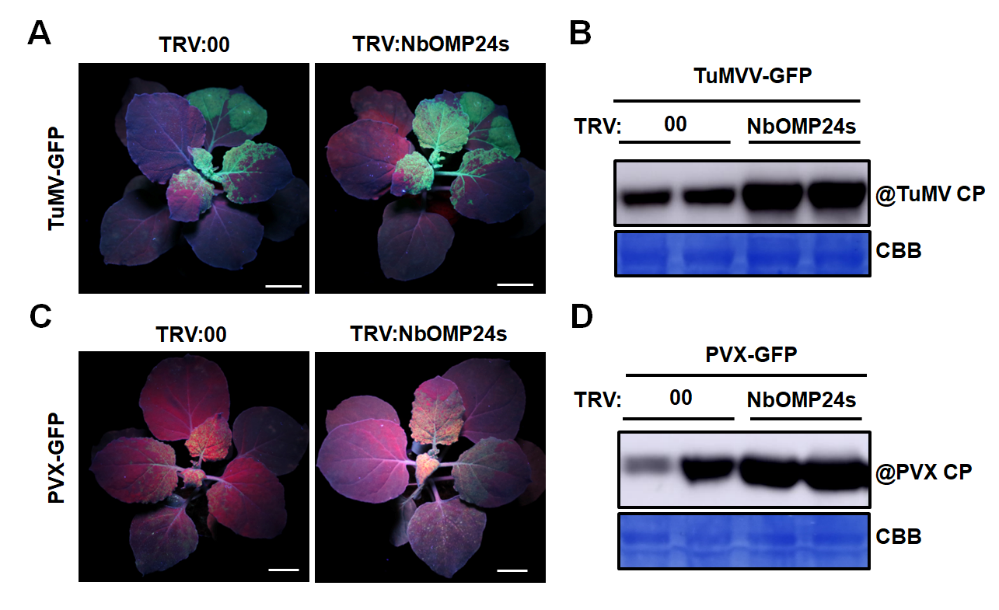


**Fig S13 Silencing of NbOMP24s facilitated TuMV and PVX infection.** (A, C) GFP fluorescence and viral symptoms of TRV:NbOMP24-treated plants then inoculated with TuMV-GFP (A) or PVX-GFP (C). Photos were taken 8 days after inoculation with TuMV-GFP (A) or PVX-GFP (C) under UV light. Bars = 2 cm. (B, D) The accumulation of TuMV CP (B) or PVX CP (D) in systemic leaves from TRV:NbOMP24 plants were more than in the controls (TRV:00) as shown by western blot.


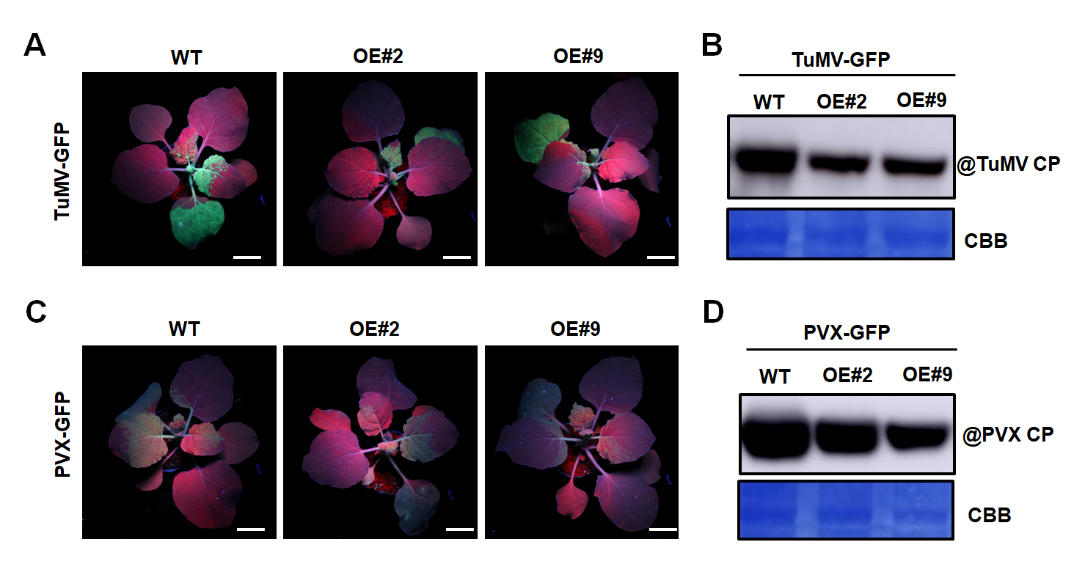


**Fig S14 NbOMP24 positively regulates resistance to two other viruses** (A, C) GFP fluorescence and viral symptoms in plants overexpressing NbOMP24.1 (OE#2 and OE#9) inoculated with TuMV-GFP (A) or PVX-GFP (C). Photos were taken 6 days after inoculation with TuMV-GFP (A) or PVX-GFP (C) under UV light. (B, D) The accumulation of TuMV CP (B) or PVX CP (D) in systemic leaves from plants overexpressing NbOMP24.1 were less than in the controls (wild type) as shown by western blot.


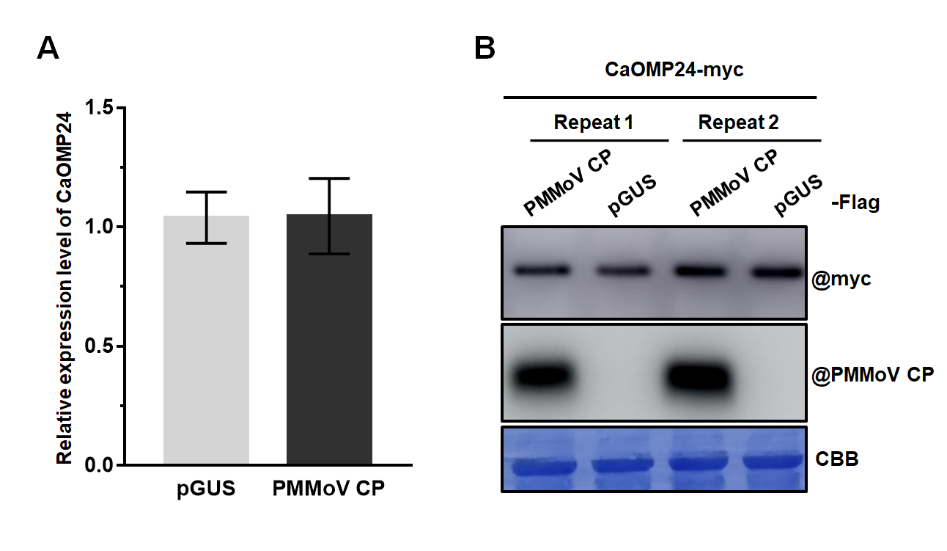


**Fig S15 The mRNA (A) and protein (B) expression levels of CaOMP24 were not affected by co-expression with PMMoV CP.**


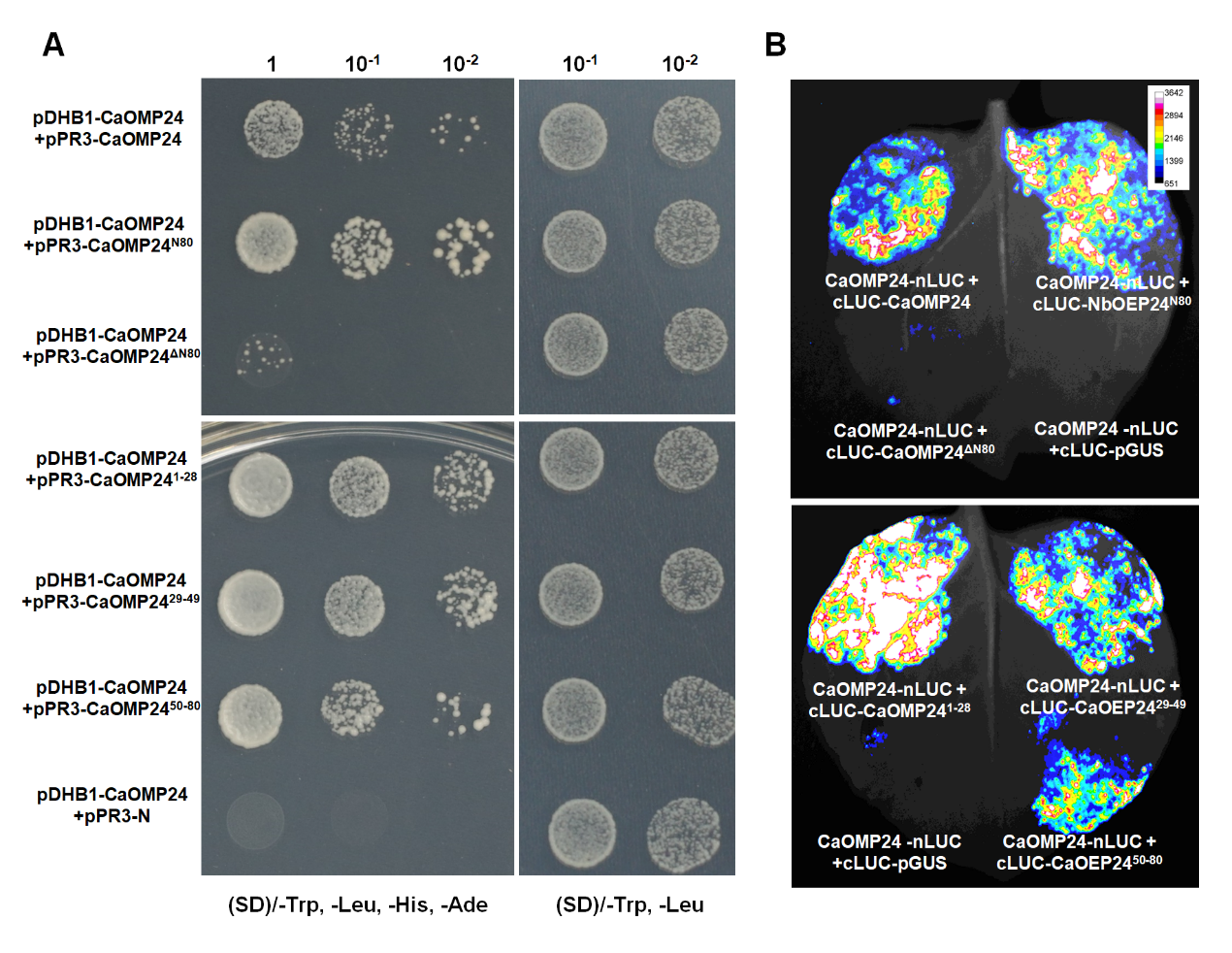


**Fig S16 Analysis of the region of CaOMP24 for its self-interaction by Y2H (A) and LCI (B).**

**
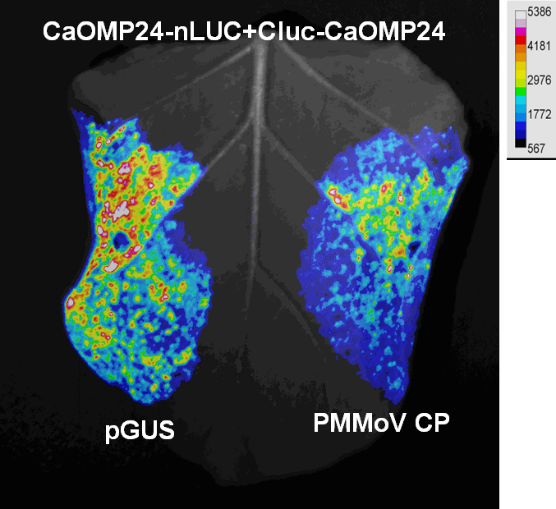
**

**Fig S17 LCI assays demonstrating that the bioluminescence signal generated by CaOMP24-nLUC/cLUC-CaOMP24 was weakened in the presence of PMMoV CP.** Agrobacterium cultures harboring CaOMP24-nLUC/cLUC-CaOMP24 were mixed with Agrobacterium harboring PMMoV CP-Flag (right) or pGUS-Flag(left), and infiltrated into a single leaf of *N. benthamiana*.


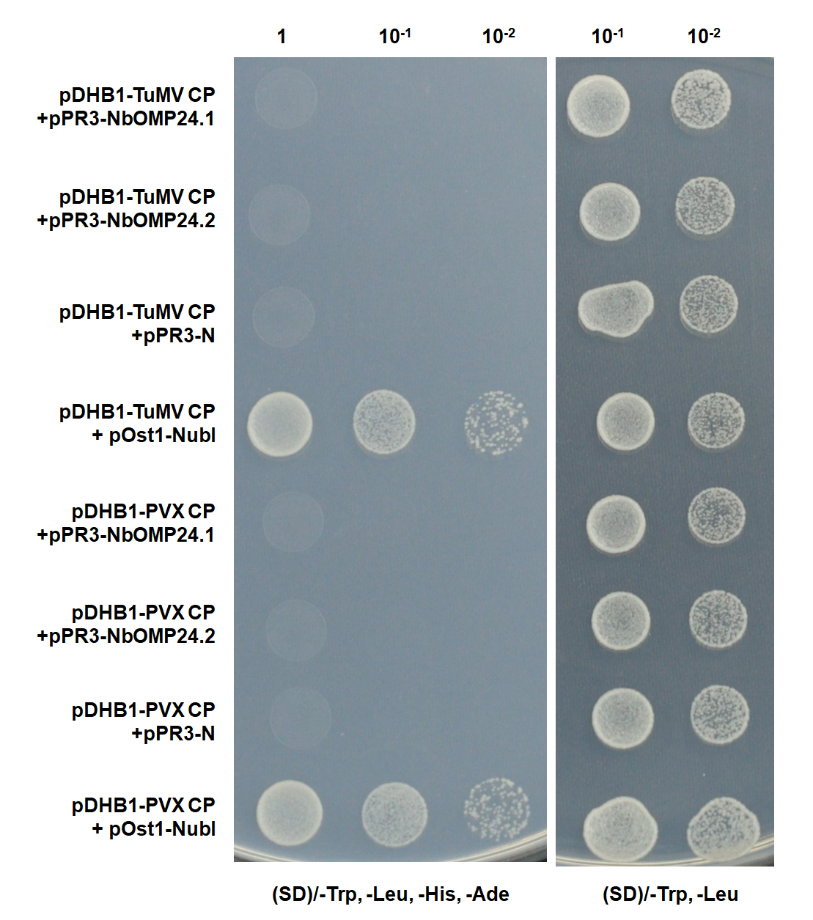


**Fig S18 Y2H assay analysis of the interactions between NbOMP24s and PVX or TuMV CP.**
